# Single-Cell Integration Analysis of Heterotopic Ossification and Fibrocartilage Developmental Lineage: Endoplasmic Reticulum Stress Effector Xbp1 Transcriptionally Regulates the Notch Signaling Pathway to Mediate Fibrocartilage Differentiation

**DOI:** 10.1155/2021/7663366

**Published:** 2021-10-26

**Authors:** Yisheng Chen, Yaying Sun, Yuzhen Xu, Wei-Wei Lin, Zhiwen Luo, Zhihua Han, Shaohua Liu, Beijie Qi, Chenyu Sun, Ken Go, x.-R. Kang, Jiwu Chen

**Affiliations:** ^1^Department of Orthopedics, Shanghai General Hospital, Shanghai Jiao Tong University School of Medicine, Shanghai Jiao Tong University, Shanghai 200080, China; ^2^Department of Sports Medicine, Huashan Hospital, Fudan University, Shanghai, China; ^3^Department of Rehabilitation, The Second Affiliated Hospital of Shandong First Medical University, Taian, Shandong Province 271000, China; ^4^Department of Neurosurgery, Second Affiliated Hospital of Zhejiang University School of Medicine, Zhejiang University, 88 Jiefang Road, Hangzhou, 310009 Zhejiang, China; ^5^Internal Medicine, AMITA Health Saint Joseph Hospital Chicago, 2900 N. Lake Shore Drive, Chicago, 60657 Illinois, USA; ^6^Department of Clinical Training Centre, St. Marianna Hospital, Tokyo, Japan; ^7^Shanghai Jiao Tong University, Shanghai 200080, China

## Abstract

**Introduction:**

Regeneration of fibrochondrocytes is essential for the healing of the tendon-bone interface (TBI), which is similar to the formation of neurogenic heterotopic ossification (HO). Through single-cell integrative analysis, this study explored the homogeneity of HO cells and fibrochondrocytes.

**Methods:**

This study integrated six datasets, namely, GSE94683, GSE144306, GSE168153, GSE138515, GSE102929, and GSE110993. The differentiation trajectory and key transcription factors (TFs) for HO occurrence were systematically analyzed by integrating single-cell RNA (scRNA) sequencing, bulk RNA sequencing, and assay of transposase accessible chromatin seq. The differential expression and enrichment pathways of TFs in heterotopically ossified tissues were identified.

**Results:**

HO that mimicked pathological cells was classified into HO1 and HO2 cell subsets. Results of the pseudo-temporal sequence analysis suggested that HO2 is a differentiated precursor cell of HO1. The analysis of integrated scRNA data revealed that ectopically ossified cells have similar transcriptional characteristics to cells in the fibrocartilaginous zone of tendons. The modified SCENIC method was used to identify specific transcriptional regulators associated with ectopic ossification. Xbp1 was defined as a common key transcriptional regulator of ectopically ossified tissues and the fibrocartilaginous zone of tendons. Subsequently, the CellPhoneDB database was completed for the cellular ligand-receptor analysis. With further pathway screening, this study is the first to propose that Xbp1 may upregulate the Notch signaling pathway through Jag1 transcription. Twenty-four microRNAs were screened and were found to be potentially associated with upregulation of XBP1 expression after acute ischemic stroke.

**Conclusion:**

A systematic analysis of the differentiation landscape and cellular homogeneity facilitated a molecular understanding of the phenotypic similarities between cells in the fibrocartilaginous region of tendon and HO cells. Furthermore, by identifying Xbp1 as a hub regulator and by conducting a ligand–receptor analysis, we propose a potential Xbp1/Jag1/Notch signaling pathway.

## 1. Introduction

Tendon to bone healing is a major concern in sports medicine. Regardless of the advances in rotator cuff repair and anterior, healing in the tendon-bone interface (TBI) is the chief problem that has attracted significant attention [1–4].

The TBI contains four coherent layers with gradual composition, structure, and mechanical properties. Histologically, the TBI is composed of bone, calcified fibrocartilage, noncalcified fibrocartilage, and tendon, which ensures the smooth transmission of force from the muscle to bone [5–8]. However, following rotator cuff repair or anterior cruciate ligament reconstruction, large amounts of fibrovascular tissue accumulate in the TBI, hindering healing and weakening the biomechanical properties of the joint [6–12]. Accumulating evidence has demonstrated that fibrochondrocytes localized within the TBI are essential for tendon to bone healing [13]. Rui et al. found that the tendon and bone healed well in rabbits with more fibrocartilage cells, and the promotion of fibrocartilage cell proliferation could enhance tendon-bone healing [8]. Therefore, the study of the origin and characteristics of fibrochondrocytes could help improve tendon to bone healing [13].

Neurogenic heterotopic ossification is a common complication of acute ischemic stroke; however, its exact mechanism remains unknown. Heterotopic ossification (HO) is the appearance of osteoblasts in the soft tissues and the formation of bone tissue [14, 15]. Interestingly, HO demonstrates several characteristics similar to the healing process of the TBI. First, the tissue in HO is histologically similar to the TBI [14–16]. As an important feature of the tissue in HO and an essential process for fibrocartilage development, mineralization is observed in both HO and TBI formation [6, 16, 17]. Second, mechanical stimulation is pivotal for the development of both TBI and HO [16, 18]. Third, bone marrow-derived mesenchymal stem cells (BM-MSCs) and tendon-derived stem cells, the cellular sources of fibrocartilage and HO, have been reported to possess a multidifferentiation potential [19–25]. Fourth, numerous studies have demonstrated that the Notch and Hedgehog (Hh) signaling pathways could drive ectopic ossification and tendon to bone healing by mediating associated transcription factors, although the exact underlying mechanisms remain unclear [20, 26–30]. Fifth, some genes, such as COL2A1 and MKX, were found to be associated with the upregulation of the Notch and Hh signaling pathways and have the potential to promote tendon to bone healing [27, 30–33]. Moreover, the transcript levels of these genes (including COL2A1 and MKX) were found to be upregulated in ectopic ossified tissues [27, 34, 35]. Therefore, considering the homogeneity between HO and the TBI, we hypothesized that research on HO might serve as the reference for a better understanding of tendon to bone healing. Exploring the key regulators and pathways associated with the development of HO might provide novel insights into fibrocartilage restoration in the TBI.

The single-cell technique was used in this study to investigate the homogeneity between HO and the TBI. The single-cell transcriptome, which is a recent, novel research method, enables comprehensive exploration of tissue microstates and thus the characterization and changes in the cellular condition [36–39]. The scRNA-seq transcriptome sample integration analysis has been used to assess cellular homology across different sequencing platforms at different centers [40, 41]. We speculate that the homogeneity between the two cells can be explored by integrating the analysis of HO tissue and fibrocartilage cells by single-cell techniques.

Transcription factors are targets for the regulation of cell differentiation [42]. In recent years, significant progress was achieved in the construction of gene regulatory networks (GRNs) based on single-cell transcriptome expression data. This data would help us understand the transcriptional regulatory networks of cells and uncover key TFs [43, 44]. On the other hand, transcription factor-mediated changes in cellular communication and pathway signaling are important manifestations of cellular homogeneity [45]. Cellular communication is defined as the transmission of information from one cell to another through a medium to produce a corresponding response. Ligand-receptor complex-mediated cell-cell communication is essential for coordinating various biological processes, such as development, differentiation, and inflammation [46]. CellPhoneDB serves as an integrated algorithm for identifying biologically relevant ligand-receptor interactions from the scRNA-seq data, which could be used to investigate new targets for disease treatment, while ligand proteins in biological signaling are regulated via transcription [47]. Numerous studies have demonstrated that ligand manipulation therapy is a viable method of targeted therapy [31]. Therefore, after constructing GRNs based on single-cell transcriptome expression data, we propose the use of that technique for ligand-receptor analysis, which may contribute to the identification of key transcription factors for cellular developmental targets in the HO and fibrocartilage regions, thus providing a theoretical foundation for future ligand therapy.

In this study, three single-cell sequencing datasets (GSE168153, GSE138515, and GSE102929) were available for the cells in the tendon fibrochondral region and HO tissue [46–48]. After the GRNs were constructed, ligand-receptor analysis was performed to identify the core transcription factors. We aimed to identify common intercellular differentiation and transcriptional regulation pathways associated with fibrocartilage development in the TBI and HO.

## 2. Materials and Methods

### 2.1. Data Retrieval

All data were obtained from the Gene Expression Omnibus database, which mainly includes the GSE94683, GSE144306, GSE168153, GSE138515, GSE102929, and GSE110993 datasets (Table 1). GSE94683 includes transcriptional profiles of mesenchymal stromal cells in ectopically ossified tissues from nine healthy donors and seven patients with HO. GSE168153 is the only single-cell sequencing dataset available for the fibrocartilaginous region of mouse tendons. GSE144306 and GSE168153 are datasets of the same study, and GSE144306 is an assay for transposase-accessible chromatin with high-throughput sequencing (ATAC-seq) dataset. GSE138515 contains single-cell transcriptome data from the Achilles tendon of 6-week-old male C57Bl/6 mice. GSE102929 reveals single-cell RNA transcriptome data from 4-week-old mouse tail tendon cells under physiological and simulated HO pathological conditions. Lactating rat tail tendon has less extracellular matrix, more number of cells per tissue volume, and strong cell growth and differentiation ability. Primary and passaged tendon cells can be obtained in large quantities relatively quickly, facilitating subsequent experiments. Therefore, GSE102929 can be used to reveal the differentiation trajectory characteristics and heterogeneity of tendon-derived cells under pathological HO conditions. GSE110993 is a dataset reflecting microRNA changes following acute ischemic stroke. This dataset was used to screen for microRNAs with altered expression in the serum after acute ischemic stroke. The target mRNAs of the miRNAs were predicted using the miRWalk database (http://mirwalk.umm.uni-heidelberg.de/).

### 2.2. Differential Expression Analysis of TFs

The TTRUST dataset is a database that records the regulatory relationships of TFs and contains not only the target genes corresponding to TFs but also the regulatory relationships among TFs [49]. It was used to screen for differentially expressed TFs in HO tissues. Differential analysis was performed by the R software limma package, and *p* values less than 0.05 were defined as significantly different. Differentially expressed genes were ranked according to |logFC|, and 70 genes that were highly expressed in HO tissues were obtained.

### 2.3. Single-Cell RNA Sequencing (RNA-seq) Analysis

RNA-seq analysis was performed using the Seurat package based on the R software (version 4.0.2) [50]. Canonical correlation analysis (CCA) was also used for dataset integration. Principal component analysis (PCA) and uniform manifold approximation and projection/*t*-distributed stochastic neighbor embedding (UMAP/TSNE) methods were used for the scRNA-seq dimensionality reduction analysis. Cell annotation of GSE168153 and GSE138515 was completed according to the information provided in the original article, and the CellMarker database and SingleR package were used for further correction [51, 52]. The “FindAllMarkers” function in Seruat was further used for the differential expression analysis, as described in the previous analysis. Monocle 2 and Monocle 3 were used to perform pseudo-temporal trajectory analysis [53]. Cellular ligand-receptor analysis was performed according to the instruction manual using the CellPhoneDB database and software [54]. Raw data of all analysis processes were saved.

### 2.4. Hub TF Analysis

To identify specific transcriptional regulators associated with ectopic ossification, a modified version of the SCENIC method was used to construct a regulatory network between HO-associated targets and TFs [55, 56]. Thus, each TF has its corresponding downstream target gene. Currently, SCENIC only supports transcriptional positive regulation analysis. Based on the above results, the regulon activity score was calculated for each cell, and the method was repeated three times to confirm the stability of the regulatory relationships [57]. Then, by performing Pearson correlation coefficient analysis for each pair of regulatory relationships, the similarity of the overall regulatory activity of different TFs was assessed, and this was used to quantify the regulatory correlations among cell types. The top 5 genes in each cell were screened for correlation analysis.

### 2.5. ATAC-seq Analysis

The Fastq sequencing data for the ATAC-seq dataset of the tendon-bone union region were obtained from GSE144306. Filtering and quality control were performed with the trim_galore software, and bowtie2 was used for comparison and calculation of statistical comparison rates. After finding peaks using MACS2, the length of the insert fragments, fraction of reads in peak values, and irreproducibility discovery rate were used to calculate duplicates. The MEME tool was then used to annotate motifs and verify potential transcriptional regulatory relationships [58].

### 2.6. Enrichment Analyses

Four main methods of enrichment analysis were used in this study, namely, Gene Set Enrichment Analysis (GSEA), Gene Set Variation Analysis (GSVA), pathway enrichment analysis, and Gene Ontology (GO) and Kyoto Encyclopedia of Genes and Genomes (KEGG) enrichment analysis: (1) GSEA was conducted using the Molecular Signatures Database (MSigDB) Collection gene set database, and the database for the reference genes was MSigDB Collections (c2.cp.v7.2. symbols.gmt) [59, 60]. (2) The GSVA for microarray and RNA-Seq dataset uses the reference gene database MSigDB Collections (2.cp.kegg.v6.2.symbols) [61]. False discovery rate < 0.25 and *p*.adjust < 0.05 were considered significantly enriched. (3) The pathway enrichment analysis of single cells was performed using the Single-Cell Signature Explorer, and the expression abundance of the target pathway is calculated. (4) GO and KEGG enrichment analyses are widely used research methods; they are conducted using the clusterProfiler package (version 3.14.3), and the org.Mm.eg.db package (version 3.10.0) was used for ID transformation [59, 62, 63].

### 2.7. Statistical Analysis and Graphing

Statistical graphs were created using the R software (version 4.0.2, R Foundation for Statistical Computing, Vienna, Austria). Comparisons between two groups were performed using a two-tailed, unpaired Student's *t*-test, and the results are presented as standard deviation ± mean. Analysis of variance was used to compare multiple groups.

## 3. Results

### 3.1. Differential Expression and Enrichment Pathways of TFs in Ectopically Ossified Tissues

**Figure 1:**
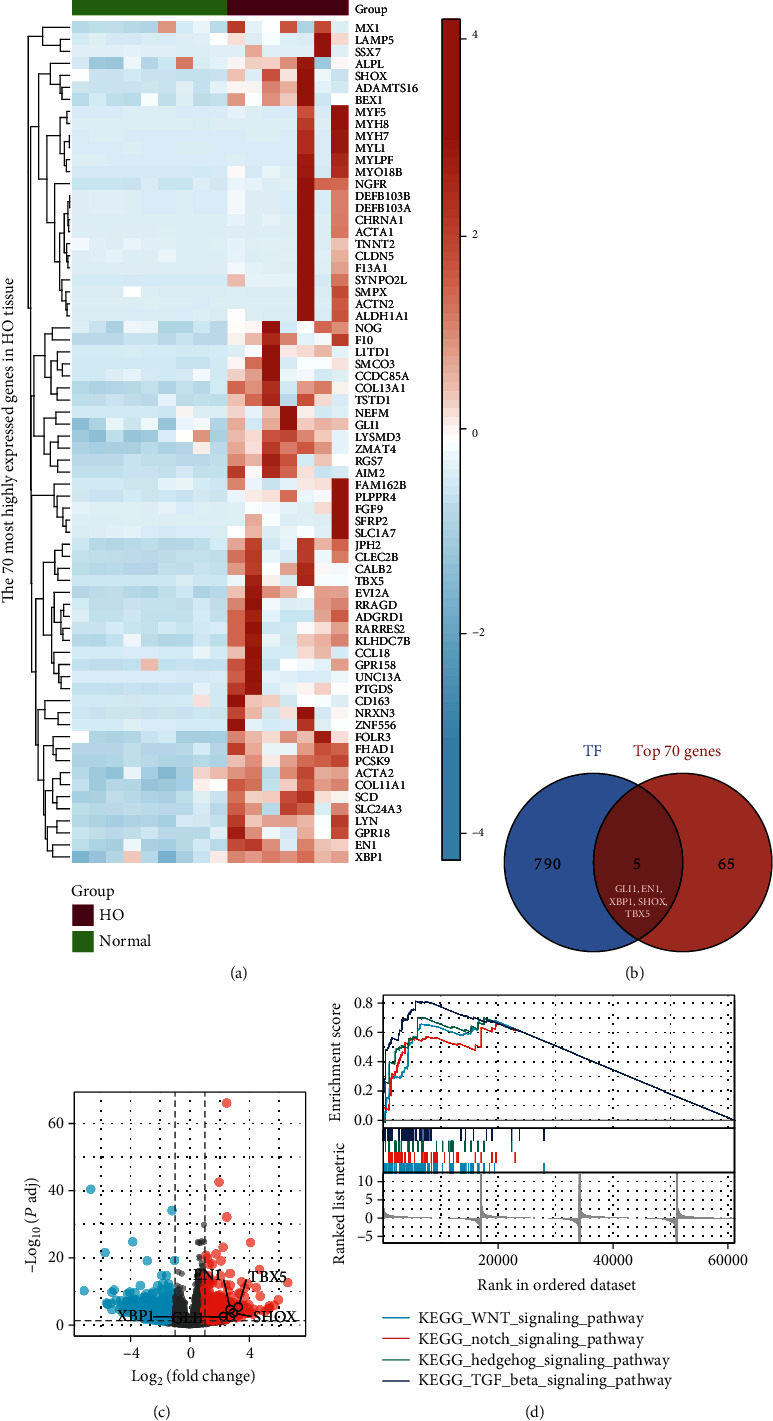
Characterization of the differential expression and enrichment pathways of transcription factors in ectopically ossified tissues. (a) Heat map of the top 70 genes expressed in heterotopic ossification (HO) tissues. (b) Intersection of the top 70 genes and 795 genes from the TTRUST database. (c) Volcano plot of five differential transcription factor expression levels based on GSE94683 data. (d) Visualization of four HO-related KEGG pathways based on GSEA results. GSEA: Gene Set Enrichment Analysis; KEGG: Kyoto Encyclopedia of Genes and Genomes.

Differential analysis was performed on 17076 genes from GSE94683 (including nine healthy donor samples and seven samples of ectopically ossified tissues from patients with HO). Among them, 1203 genes satisfied the threshold of ∣log2(FC) | >1 and *p*.adj < 0.05, 456 genes were highly expressed in HO tissues, and 747 genes were lowly expressed in HO tissues. Then, 70 genes with the highest expression in HO tissues were screened, and the differential expressions of these genes were visualized in different samples by drawing a heat map (Figure 1(a)). Moreover, 795 TFs identified were obtained from the TTRUST database. By plotting Venn diagrams, five genes, namely, GLI1, EN1, XBP1, SHOX, and TBX5, were found to be the intersection of the 795 TFs and 70 genes highly expressed in HO tissues (Figure 1(b)). The characteristics of these five TFs, namely, GLI1, EN1, XBP1, SHOX, and TBX5, differentially expressed in GSE94683 are shown in Figure 1(c). Thus, to explore the biological pathways differentially expressed in HO tissues, we performed GSEA. The analysis suggested that the WNT, Notch, Hh, and transforming growth factor (TGF) beta signaling pathways in the KEGG pathway were highly expressed in HO tissues (*p* < 0.05).

### 3.2. Heterogeneity and Proposed Temporal Differentiation Trajectory of an Ectopic Ossification Cell Model

**Figure 2:**
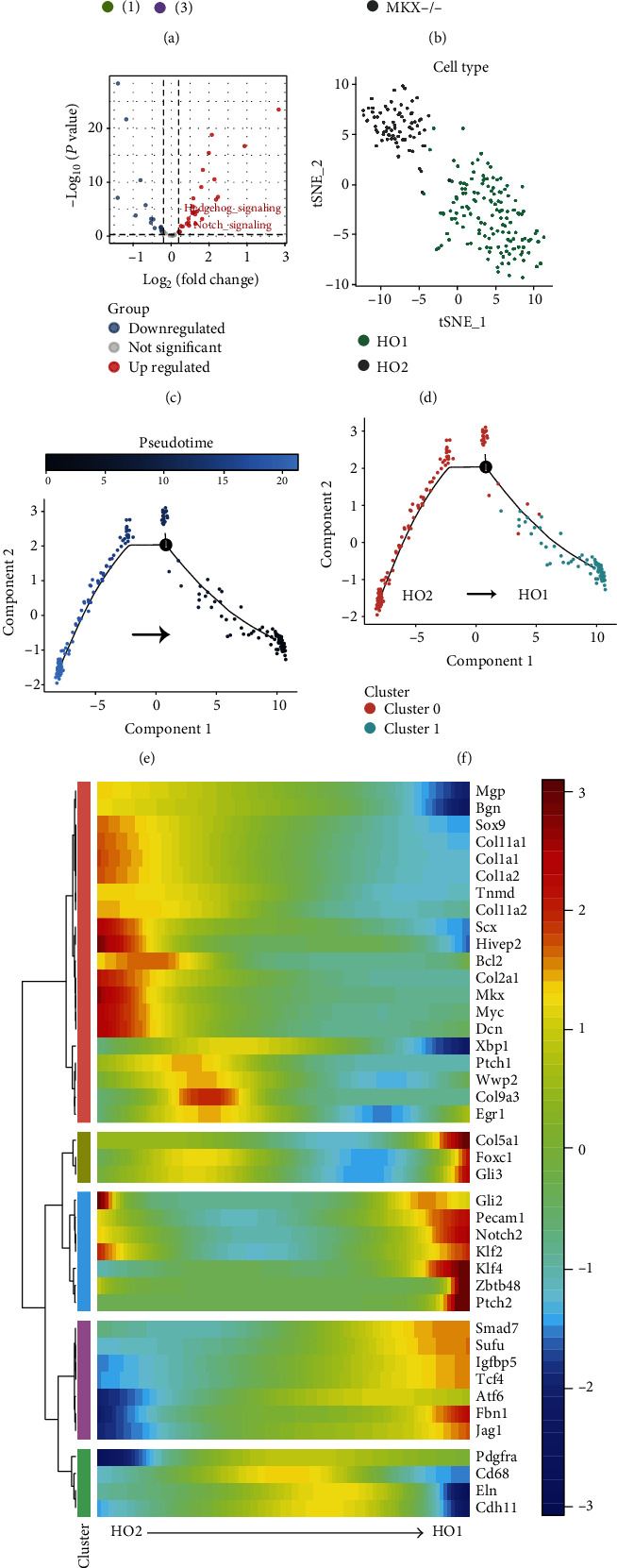
Characterization of the heterogeneity and pseudo-temporal differentiation trajectories in heterotopic ossification (HO) cell models. (a) tSNE subcluster clustering maps of mouse tail tendon cells under physiological and simulated HO pathological conditions, with different clusters indicated by colors. (b) tSNE subcluster clustering maps of mouse tail tendon cells under physiological and simulated HO pathological conditions, with green and gray indicating tail tendon cells under physiological conditions and simulated HO pathological conditions, respectively. (c) Volcano map of the pathway differential expression in mouse tail tendon cells under physiological and simulated HO pathological conditions. (d) Uniform manifold approximation and projection (UMAP) clustering of mouse tail tendon cells under simulated HO pathological conditions, with HO1 class cells (cluster 0) defined in green and HO2 class cells (cluster 1) defined in gray. (e, f) Pseudo-temporal sequence of HO cell differentiation in mice. (g) Expression of HO-related genes in mouse tail tendon cells under simulated HO pathological conditions with the change of differentiation chronology.

At present, the differentiation trajectory of ectopically ossified cells remains unknown, and the associated single-cell transcriptome has not been fully characterized. Silencing Mkx is shown as a viable method of constructing ectopic ossification models [15, 64, 65]. GSE102929 reveals scRNA transcriptomic data from 4-week-old mouse tail tendon cells under physiological and simulated HO pathological conditions, all filtered, normalized, and clustered. UMAP clustering analysis of single-cell RNA-seq revealed that single cells of mouse tail tendon cells could be distinguished into four clusters under physiological and simulated HO pathological conditions (Figure 2(a)). These cell clusters were found to correlate with the cell model groups. Among them, clusters 0 and 3 belong to the scRNA-seq data of mouse tail tendon cells under physiological conditions, while clusters 1 and 2 belong to the scRNA-seq data of mouse tail tendon cells under pathological conditions (Figure 2(b)). Furthermore, GSVA was used to quantify pathway activity within every single cell, all differential pathways (*p* < 0.05) were extracted, and a pathway heat map (Supplementary Figure 1A) was drawn according to the pathway activity of each sample. Cluster 2 has a more pronounced degree of HO differentiation than cluster 1. Therefore, the pathway activities of clusters 2 and 0 were compared to further identify the pathways that were significantly upregulated/downregulated in ectopic ossified tissues. Compared with the control group (cluster 0), the myogenic MYOGENESIS and MYC TARGETS pathways were lowly expressed in the ectopic ossification model group (cluster 2), and KRAS SIGNALING DN was also lowly expressed in cluster 2 (Supplementary Figure 1B-1D). By contrast, the ectopic ossification-related pathways, namely, Hh and Notch signaling pathways, were highly expressed, and the oxidative phosphorylation pathway was also highly expressed in cluster 2 (Supplementary Figure 1E-1G). Mkx was significantly upregulated in cluster 4 (Supplementary Figure 2A, 2B). Then, heat maps were plotted to further demonstrate the degree of differential expression between the Notch and the Hh signaling pathways (Figure 2(c)).

Ectopic ossification mimicking pathological cells were reclustered and defined as HO1 (corresponding to Supplementary Figure 2C, cluster 1) and HO2 (corresponding to Supplementary Figure 2C, cluster 0) (Figure 2(d) and Supplementary Figure 2C). The results of the mimetic timing analysis suggested that HO2 was a precursor cell for HO1 differentiation (Figures 2(e) and 2(f)). To further characterize the pathological differentiation of ectopically ossified cells, the proposed chronological expression profile of genes associated with the ectopic ossification phenotype was revealed (Figure 2(g)). The gene expression profiles of Xbp1, E2f7, Fgfr1, Icam1, Jag1, and Ncam1 are shown in Supplementary Figure 2D. Mgp, Bgn, Sox9, Col11a1, Col1a1, Col1a2, Tnmd, Col11a2, Scx, Hivep2, Bcl2, Col2a1, Mkx, Myc, and Dcn were enriched in HO2-like cells. Col5a1, Foxc1, Gli3, Gli2, Pecam1, Klf2, Klf4, Zbtb48, Ptch2, Smad7, Sufu, Igfbp5, Tcf, Fbn1, and Jag1 were enriched mainly in HO1-like cells (Figure 2(g)). Among them, Gli3, Cli2, Pecam1, and Ptch2 are the star genes of the Hh signaling pathway, and Notch2 and Jag1 are the star genes of the Notch signaling pathway, which suggested that the activities of the Notch and Hh signaling pathways may be upregulated in HO1-like cells. According to the expression characteristics of these genes, HO1-like cells are more similar to chondrocyte-like cells, and HO2-like cells are more similar to tenocyte-like cells. Therefore, HO2 cells were defined as “tenocyte-like cells.”

### 3.3. Endoplasmic Reticulum (ER) Stress Effector XBP1 Was Found to Be a Key Transcriptional Regulator in Ectopically Ossified Tissues

**Figure 3:**
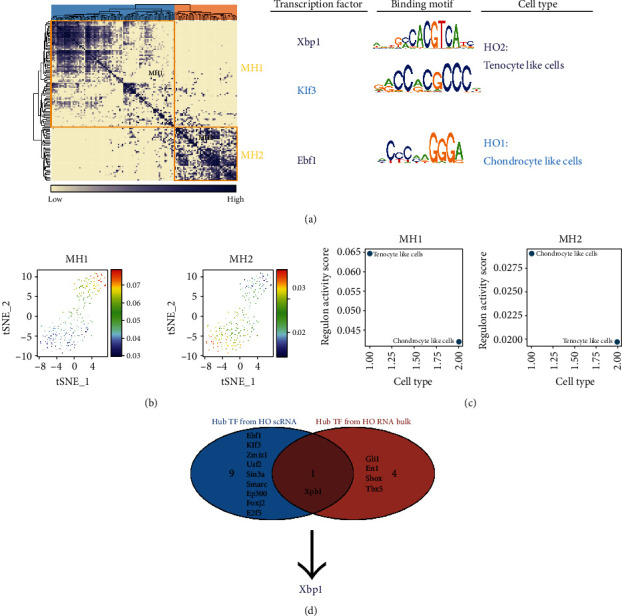
Endoplasmic reticulum stress effector XBP1 was found to be a key transcriptional regulator in ectopic ossified tissues. (a) Regulatory modules were identified based on the regulon connection specificity index matrix, and the core transcription factors, binding motifs, and corresponding cell types were extracted. (b) The activity of each module in different cells is represented by tSNE plots. (c) Corresponding cell types of different modules. (d) The Venn diagram reveals that Xbp1 is a common key transcription factor for heterotopic ossification bulk-RNA-seq and scRNA-seq analyses.

In this study, key TFs in HO1-like cells (chondrocyte-like cells) and HO2-like cells (tenocyte-like cells) were identified using comprehensive network analysis (Supplementary Figure 3). The network analysis identified Ebf1, Usf2, Klf3, Zmiz1, and Sin3a as key transcriptional regulators in HO1-like cells, and Ep300, Smarcc, Foxj2, Xbp1, and E2f5 are key transcriptional regulators in HO2-like cells. To systematically compare the transcriptional profiles in different cells, we compared the expression correlation of each TF based on the connection specificity index (CSI) (Figure 3(a)). In HO1-like cells (chondrocyte-like cells) and HO2-like cells (tenocyte-like cells), Xbp1 and Klf3 were found to be key transcriptional regulators of MH1 module cells, and TF Ebf1 was found to be a key transcriptional regulator of MH2 module cells. In addition, the MH1 module was found to be more active in HO2-like (tenocyte-like cells) cells, while the MH2 module was more active in HO1-like (chondrocyte-like cells) cells (Figures 3(b) and 3(c)). This also demonstrates that Xbp1 and Klf3 are key transcriptional regulators in HO2 (tenocyte-like cells), while Ebf1 is a key transcriptional regulator in HO1 (chondrocyte-like cells). The Venn diagram reveals that Xbp1 is a common key TF for HO tissue bulk-RNA-seq and scRNA-seq analyses (Figure 3(d)).

### 3.4. Ectopically Ossified Cells Have Similar Transcriptional Characteristics to Cells in the Fibrocartilaginous Zone of Tendons

Based on the proposed temporal analysis characteristics of the gene expression in Figure 2(g), combined with the results of previous studies, the gene expression characteristics of ectopic ossification demonstrated strong similarities to those of the fibrocartilaginous region of the tendon-bone union [8, 66–68]. Therefore, we hypothesized that ectopically ossified cells have similar transcriptional characteristics to cells in the fibrocartilaginous zone of the tendon-bone junction region. Then, we integrated scRNA-seq transcriptome data from mouse Achilles tendon, scRNA-seq transcriptome data from mouse tendon junction region, and scRNA-seq transcriptome data from mouse tail tendon cells under simulated HO pathological conditions for scRNA-seq integration analysis.

**Figure 4:**
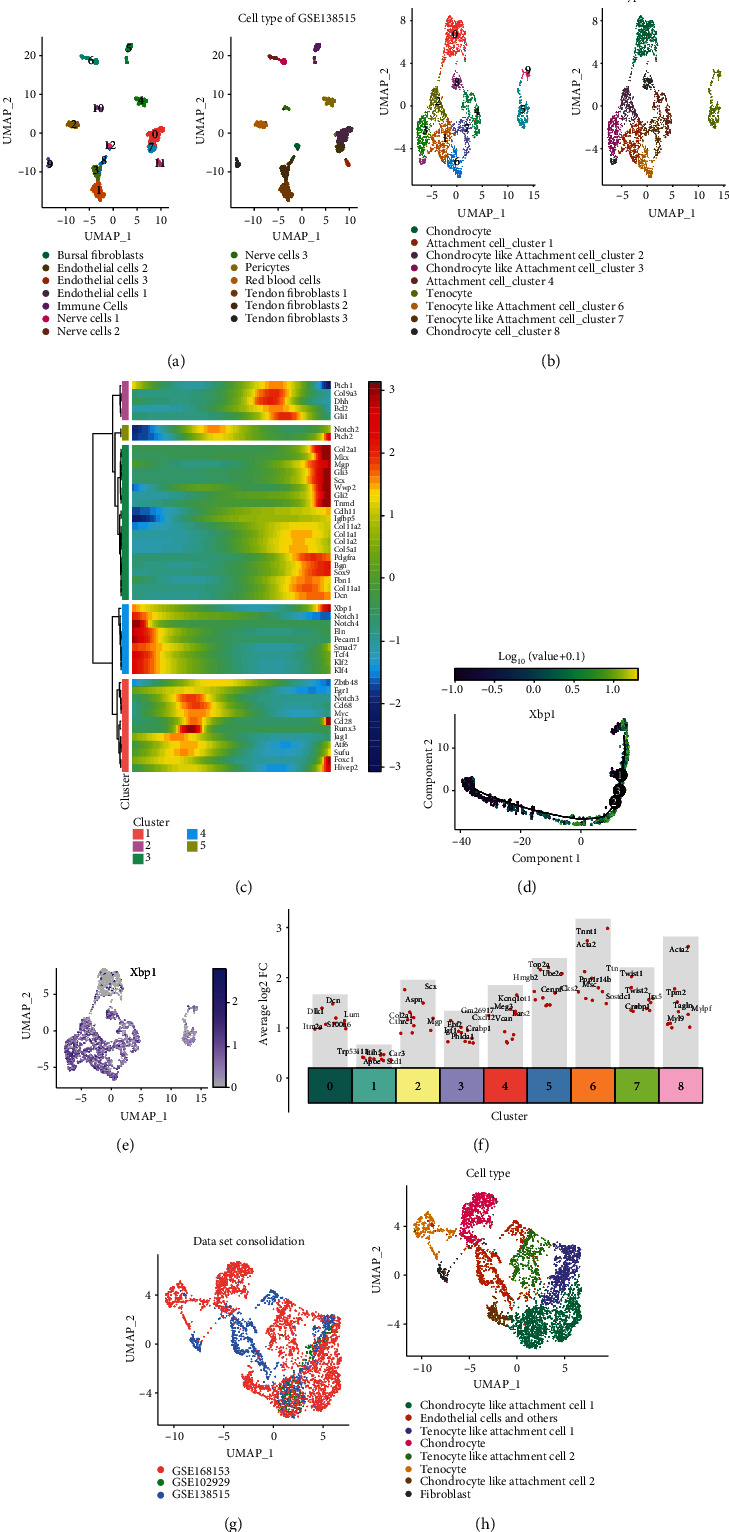
Ectopically ossified cells have similar transcriptional characteristics to cells in the tendon fibrocartilaginous region. (a) For GSE138515, cell clustering and annotation are shown on the uniform manifold approximation and projection (UMAP) plots. (b) For GSE168153, cell clustering and annotation are presented on the UMAP plots. (c) For GSE168153, the key gene expression features of ectopic ossification and the tendon fibrocartilaginous zone are presented with a pseudo-temporal analysis. (d) Expression features of Xbp1 on pseudo-temporal trajectories. (e) Expression characteristics of Xbp1 in different cells in GSE168153 shown on the UMAP plots. (f) Differential gene expression analysis of the top 5 genes showing upregulated expression in all eight clusters in the integrated dataset (including GSE168153, GSE138515, and GSE102929 under simulated HO pathological conditions). (g) UMAP plots showing the distribution of the different datasets. (h) Cellular annotations for the integrated datasets are shown on the UMAP plots, with different colors representing cell types.

First, the cell types of single-cell transcriptome data were annotated for GSE138515 and GSE168153. GSE138515 contains single-cell transcriptome data from the Achilles tendon of 6-week-old male C57Bl/6 mice, and PCA grouping was performed after quality control of these cells (Supplementary Figure 4A, 4C). With the UMAP method, GSE138515 was found to have 12 clusters of cell types (Figure 4(a) and Supplementary Figure 4B). The cell differentiation trajectory of GSE138515 is shown in Supplementary Figure 4D. A preliminary annotation of these cells was performed based on SingleR (Supplementary Figure 4E). Among the cell clusters, clusters 0, 7, and 11 were initially classified as endothelial cells; cluster 2 was initially classified as erythrocytes; clusters 1, 3, 4, 8, 9, 10, and 12 were initially classified as fibroblasts; cluster 5 was tentatively classified as macrophages; cluster 6 was tentatively classified as oligodendrocytes. In addition, these cells were further annotated regarding the results of previous studies (Figure 4(a)) [69]. As shown above, the quality of scRNA-seq data of GSE168153 was controlled and grouped into nine clusters (Supplementary Figure 5A, 5D). The cell differentiation trajectory of GSE168153 is shown in Supplemental Figure 5B. Moreover, these cells were further annotated based on the results of previous studies (Figure 4(b)) [66]. Accordingly, the proposed temporal expression profile of genes associated with the phenotypic cartilaginous region of tendon fibers is shown in Figure 4(c). The cell annotation method combines the results of the pseudo-temporal analysis to classify cells into tenocyte, tenocyte-like attachment cell, attachment cell, chondrocyte-like attachment cell, and chondrocyte, which are the five major cell clusters (Figure 4(c) and Supplementary Figure 5C). The trajectory of cell differentiation is presented in the following order: tenocytes, tenocyte-like attachment cells, attachment cells, chondrocyte-like attachment cells, and chondrocytes (Supplementary Figure 5C). To the best of our knowledge, this study provided the most detailed subcellular classification of the tendon junctional area to date. Moreover, this classification demonstrates the spatial distribution characteristics of cells in the fibrocartilage layer. Previous analyses identified Xbp1 as a common key TF for HO tissue bulk-RNA-seq and scRNA-seq analyses. In the present study, the results of the pseudo-temporal analysis suggested that Xbp1 was enriched in tenocytes, tenocyte-like attachment cells, and attachment cells. “Chondrocyte-like attachment cells” and “Chondrocytes” were less expressed (Figures 4(d) and 4(e)).

CCA was also used for dataset integration. Three datasets (including GSE168153, GSE138515, and GSE102929 under simulated HO pathological conditions) were integrated using the CCA approach. These datasets were then grouped into nine cell clusters (Supplementary Figure 6A, 6B). The top 5 marker genes in each cell cluster were flagged (Figure 4(f) and Supplementary Figure 6B). In addition, the UMAP graph was used to show all cell clusters, and the original annotations of all cells were also identified (Supplementary Figure 6C).  Figure 4(g) shows the distribution of datasets in the UMAP plot. Before reannotating the cells, HO2 was found to be enriched in attachment cell cluster 1 and tendon fibroblasts, while HO1 was enriched near chondrocyte-like attachment cell 3 (Figure 4(g) and Supplementary Figure 6C). After reannotation of the cells, HO2 was found to be enriched in tenocyte-like attachment cell 1, while HO1 was enriched in chondrocyte-like attachment cell 1 (Figure 4(h)). This suggests that ectopically ossified cells have similar transcriptional characteristics to cells in the fibrocartilaginous zone.

### 3.5. CCA Integration of Pseudo-Temporal Differentiation Trajectories and Gene Expression Signatures in scRNA-seq

**Figure 5:**
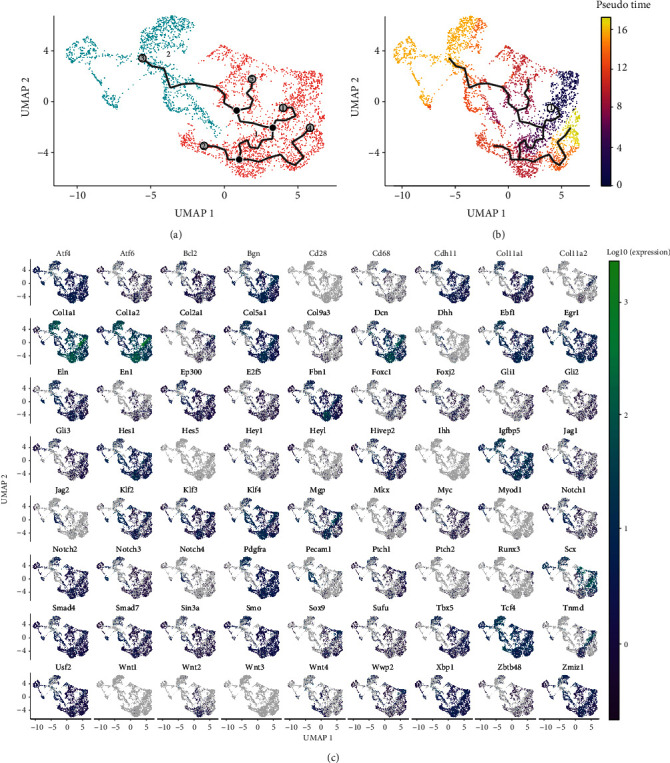
Pseudo-temporal differentiation trajectories and gene expression characteristics of the canonical correlation analysis- (CCA-) integrated single-cell datasets. (a) Pseudo-temporal series analysis of the CCA-integrated single-cell dataset based on Monocle 3. (b) Different pseudo-temporal sequences are represented by different colors in the CCA-integrated single-cell dataset. (c) Based on single-cell gene expression profiles, genes related to ectopic ossification and tendon fibrochondral region that were significant in both tenocyte-like attachment cell 1 and chondrocyte-like attachment cell 1 were significantly enriched.

The cell differentiation trajectory of mouse tail tendon cells under simulated HO pathological conditions started from HO2 to HO1 (Figures 2(e) and 2(f)). To verify that the cell differentiation trajectory of the CCA-integrated single-cell dataset is similar to that of mouse tail tendon cells under simulated HO pathological conditions, a pseudo-temporal analysis of the CCA-integrated single-cell dataset was performed based on Monocle 3 (Figure 5(a)). In the CCA-integrated single-cell dataset, the differentiation trajectory of cells started from tenocyte-like attachment cell 1 to chondrocyte-like attachment cell 1 (Figures 4(h) and 5(b)). In this study, the genes associated with ectopic ossification and the cartilage region of tendon fibers (Atf4, Bgn, Cdh11, Col11a1, Col1a1, Col1a2, Col5a1, Dcn, Eln, Ep300, Fbn1, Gli3, Hes1, Hivep2, Klf2, Klf3, Klf4, Mgp, Notch2, Pdgfra, Smad4, Smad7, Smo, Tbx5, Tcf4, Tnmd, Usf2, Xbp1, Zmiz1, etc.) were found in tenocyte-like attachment cell 1 and chondrocyte-like attachment cell 1. “Chondrocyte-like attachment cell 1” was significantly enriched in “tenocyte-like attachment cell 1” and “chondrocyte-like attachment cell 1.” The expression profiles of the key genes of ectopic ossification and tendon fibrochondral regions in the CCA-integrated single-cell dataset were presented by the pseudo-temporal analysis. *Atf4*, *Bcl2*, *Bgn*, *Cdh11*, *Col11a1*, *Col11a2*, *Col2a1*, *Col5a1*, *Dcn*, *Foxc1*, *Hes1*, *Hivep2*, *Klf3*, *Klf4*, *Mgp*, *Mkx*, *Smad4*, *Sox9*, *Tnmd*, *Xbp1*, and *Zmiz1* had high expression in the early stages of the pseudo-temporal distribution and low expression in the late stages.

### 3.6. Heterogeneity Analysis of Tenocyte-Like Attachment Cell 1 and Chondrocyte-Like Attachment Cell 1

**Figure 6:**
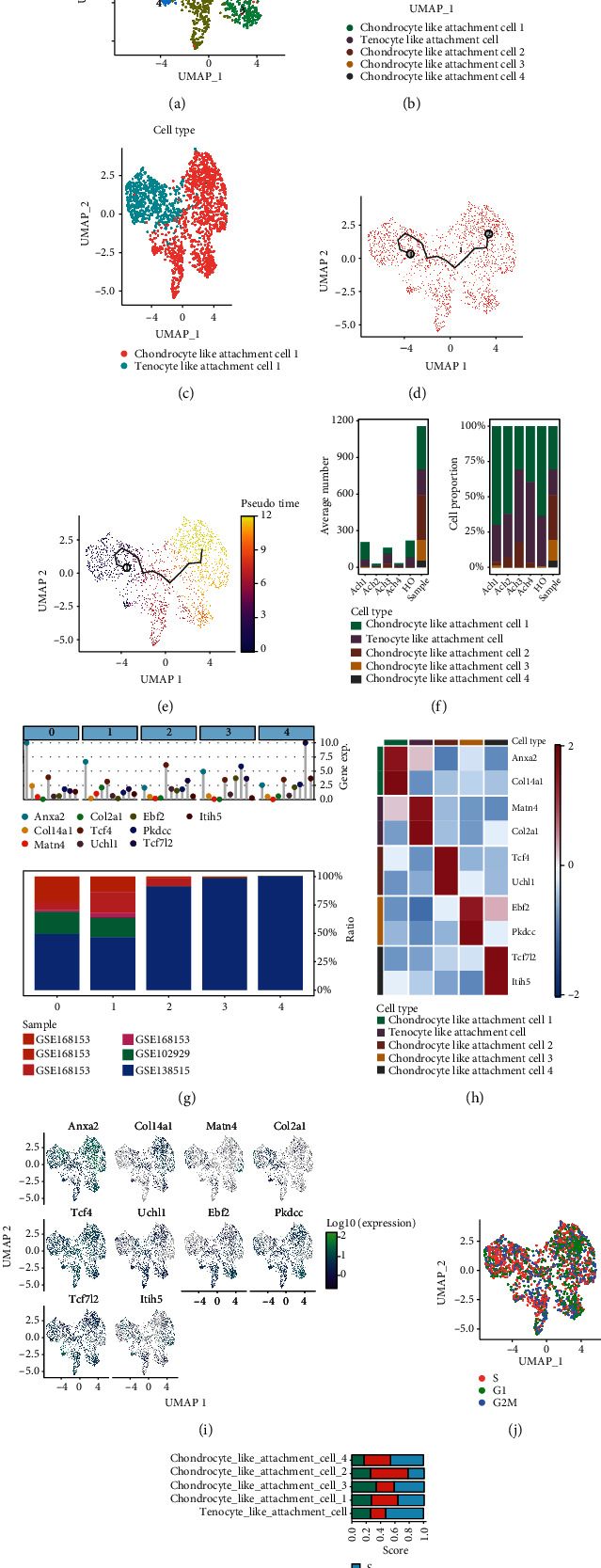
Heterogeneity analysis of tenocyte-like attachment cell 1 and chondrocyte-like attachment cell 1. (a) Uniform manifold approximation and projection- (UMAP-) based analysis of tenocyte-like attachment cell 1 and chondrocyte-like attachment cell 1 that were classified into five clusters. (b) Cell annotation map based on UMAP-classified cell clusters, namely, tenocyte-like attachment cell and chondrocyte-like attachment cells 1–4. (c) A UMAP diagram shows the correspondence of these cells with the annotated subgroups of Figure 4(h) cells. Blue indicates tenocyte-like attachment cell 1, and red indicates chondrocyte-like attachment cell 1. (d) Pseudo-temporal series analysis of this single-cell dataset based on Monocle 3. (e) Different degrees of pseudo-temporal differentiation sequences in this dataset are indicated by different colors. (f) Relative proportions and average cell counts of cell subpopulations in each tissue. (g) Core marker genes and sample preferences for each cluster. (h) Heat map of the relative expression characteristics of marker genes associated with each cell subpopulation. (i) UMAP plots of the expression characteristics of marker genes associated with each cell subpopulation. (j) The cell division cycle of all cells was explored with the Seruat package. Note: red indicates the S phase, green indicates the G1 phase, and blue indicates the G2/M phase. (k) Bar graphs of the heterogeneity of cell mitosis in each cell subpopulation.

The CCA-integrated pseudo-temporal differentiation trajectory and gene expression characterization of the single-cell dataset presented above revealed that HO cells were mainly concentrated in tenocyte-like attachment cell 1 and chondrocyte-like attachment cell 1. To demonstrate that ectopically ossified cells have similar transcriptional characteristics to cells in the fibrocartilaginous zone of the tendon-bone union, it is necessary to further confirm the transcriptional characteristics of cells from different samples in tenocyte-like attachment cell 1 and chondrocyte-like attachment cell 1. Therefore, the cells belonging to tenocyte-like attachment cell 1 and chondrocyte-like attachment cell 1 were extracted in each sample separately, a new PCA clustering analysis was performed, and five PCs were finally identified (Supplementary Figure 7A and Figure 6(a)). The first 10 marker genes of each cell cluster were marked (Supplementary Figure 7B). Marker genes of PC1 and PC2 are shown in Supplementary Figure 7C. Based on the cell annotation map of UMAP, the cell clusters were divided into the following five clusters: tenocyte-like attachment cells and chondrocyte-like attachment cells 1-4 (Figure 6(b)). Moreover, the distribution of cells from different sources is shown in Supplementary Figure 7D. The correspondence of these cells with the annotated subpopulation of cells in Figure 4(h) is also shown in Figure 6(c). Most of the cells in the tenocyte-like attachment cell 1 group in Figure 4(h) belonged to the tenocyte-like attachment cell 1 group in Figure 6(b) annotation, but a few cells belonged to chondrocyte-like attachment cell 2 cluster. The pseudo-temporal analysis of this single-cell dataset is shown in Figures 6(d) and 6(e), which suggests a cell differentiation trajectory from tenocyte-like attachment cell to chondrocyte-like attachment cell 2 and finally to chondrocyte-like attachment cell 1. This finding also suggests that the differentiation process of tenocyte-like attachment cell to chondrocyte-like attachment cell 1 is gradually progressive. The chondrocyte-like attachment cell 2 stage is the intermediate stage of the differentiation process.

For the review of cell fractionation, we found the relative proportions of the cell subpopulations, and the average cell numbers in each tissue are shown in Figure 6(f). Tenocyte-like attachment cells and chondrocyte-like attachment cells 1 and 2 were found to be the major cell types in tendon tissues and mouse tail tendon cells under simulated HO pathological conditions. The core marker genes and sample preferences for each cluster are shown in Figure 6(g). *Anxa2* and *Col14a1* are marker genes for chondrocyte-like attachment cell 1, *Matn4* and *Col2a1* are marker genes for tenocyte-like attachment cell, *Tcf4* and *Uchl1* are marker genes for chondrocyte-like attachment cell 2, *Ebf2* and *Pkdcc* are marker genes of chondrocyte-like attachment cell 3, and *Tcf712* and *Itih5* are marker genes of chondrocyte-like attachment cell 4. The expression characteristics of these cell subpopulation-associated marker genes are shown in Figure 6(i). Then, the cell division cycles of all cells were explored using the Seruat package, and more significant heterogeneity in the mitotic cell divisions of each cell subpopulation was found (Figures 6(j) and 6(k)). These series of single-cell gene expression profiles were also provided, systematically demonstrating the gene expression profile of the fibrocartilaginous region of ectopically ossified tissues and tendons (Supplementary Figure 8A). Information on the altered expression of HO-related genes with pseudo-temporal changes is shown in Supplementary Figure 8B. These cell differentiation trajectories further validate that ectopically ossified cells have similar transcriptional characteristics to cells in the fibrocartilaginous region of tendons.

### 3.7. XBP1 Is a Key Cotranscriptional Regulator in the Tendon-Bone Union Region and Ectopically Ossified Cells

**Figure 7:**
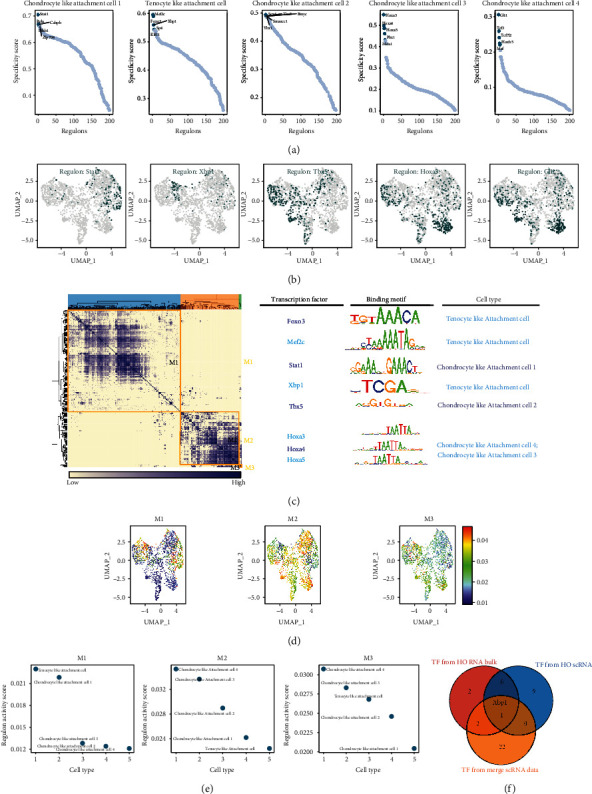
XBP1 was found to be a key cotranscriptional regulator in the fibrocartilaginous region of the tendons and ectopic ossified cells. (a) Analysis of transcription factor activities specific to different cell types in the tendon-bone union zone and ectopic ossified cells. The five most transcriptionally active transcription factors were screened for each group of cells. (b) Uniform manifold approximation and projection (UMAP) plots demonstrate the expression characteristics of the core transcription factors in each cell group. (c) Regulatory modules were identified based on the regulon CSI matrix, and the core transcription factors, binding motifs, and corresponding cell types were extracted. (d) The activity of each module in different cells is represented by UMAP plots. (e) Cell types corresponding to the different modules. (f) The Venn diagram reveals that Xbp1 is the common key transcription factor for heterotopic ossification (HO) bulk-RNA-seq, HO scRNA-seq, and the merge scRNA data.

TFs largely determine the characteristics of cell differentiation trajectories, so finding key TFs can help us explore the core mechanisms of ectopic ossification and differentiation. First, key TFs were identified in five clusters (tenocyte-like attachment cells, chondrocyte-like attachment cells 1-4) using a comprehensive network analysis combined with a modified SCENIC approach (Figure 7(a)). The key TFs in the tenocyte-like attachment cell cluster include Mef2c, Foxo3, Xbp1, Sp4, and Klf11. The key TFs in chondrocyte-like attachment cell 1 were Stat1, Reta, Cebpb, Nfkb1, and Zfp704. The key TFs in chondrocyte-like attachment cell 2 were Sox4, Tbx5, Bmyc, Smarcc1, and Ybx1. The key TFs in chondrocyte-like attachment cell 3 include Hoxa3, Hoxa4, Hoxa5, Pbx1, and Meis. The key TFs in chondrocyte-like attachment cell 4 were Gli1, Tcf3, Tcf712, Hoxb5, and Maf. The expression characteristics of some core TFs for each cell group are shown in Figure 7(b). The regulatory modules were identified based on the regulon CSI matrix, and the core TFs, binding motifs, and corresponding cell types were extracted (Figure 7(c)). The M1 module mainly included the two cell types: attachment cells and chondrocyte-like attachment cell 1 (Figures 7(d) and 7(e)). This reflects the similarity in the transcriptional regulation between tenocyte-like attachment cell and chondrocyte-like attachment cell 1. The M2 and M3 modules mainly included chondrocyte-like attachment cell 4, chondrocyte-like attachment cell 3, and chondrocyte-like attachment cell 2. The M2 and M3 modules mainly included chondrocyte-like attachment cell clusters 3 and 4 (Figures 7(d) and 7(e)). By contrast, chondrocyte-like attachment cell 2 did not exhibit module specificity. The three core TFs of the M1 module (i.e., Foxo3, Mef2c, and Xbp1) are tenocyte-like attachment cell. Stat1 is the core TF of chondrocyte-like attachment cell 1, and Tbx5 is the core TF of chondrocyte-like attachment cell 2. Intersection analysis of these key TFs with previously studied TFs revealed that TF Xbp1 was again found to be a common key TF for HO bulk-RNA-seq, HO scRNA-seq, and the integrated scRNA data.

**Figure 8:**
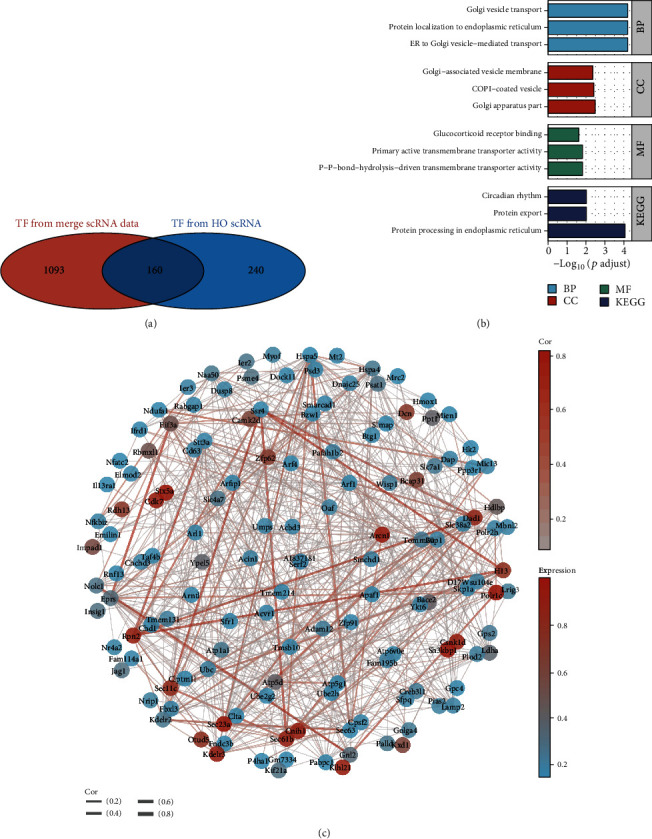
Enrichment analysis of downstream genes regulated by XBP1 with protein-protein interaction (PPI) network analysis. (a) Screening of 160 genes that were jointly used as XBP1 regulatory targets in the integrated scRNA data and heterotopic ossification (HO) scRNA data using the Venn diagram. (b) GO and KEGG enrichment analysis for XBP1-regulated genes. (c) PPI network analysis of XBP1-regulated downstream genes.

To explore the potential regulatory function of Xbp1 in the onset of ectopic ossification, the Venn diagram was used to screen 160 genes that collectively serve as downstream regulatory targets of XBP1 in the integrated scRNA data and HO scRNA data (Figure 8(a)). These 160 genes were subjected to GO and KEGG enrichment analysis (Figure 8(b)). GO enrichment analysis included three modules, namely, biological process (BP), cell component (CC), and molecular function (MF). The Golgi vesicle transport, protein localization to the ER, and ER to Golgi vesicle-mediated transport were enriched in BP; the Golgi-associated vesicle membrane, COPI-coated vesicle, and Golgi apparatus part were enriched in CC, and the glucocorticoid receptor binding, primary active transmembrane transporter activity, and P-P-bond-hydrolysis-driven transmembrane transporter activity were enriched in MF. The pathways enriched in the KEGG were the circadian rhythm, protein export, and protein processing in the ER. In summary, genes that were downstream regulatory targets of Xbp1 were found to play important roles mainly in Golgi vesicle transport, ER protein processing, cellular communication, and transport. To visualize the interaction relationships of Xbp1 downstream regulatory target genes, string sites were used to map the protein-protein interaction (PPI) network analysis of XBP1 downstream regulatory genes.

### 3.8. Ligand-Receptor and ATAC-seq Analyses Reveal That Xbp1 Upregulate the Notch Signaling Pathway by Promoting Jag1 Transcription

**Figure 9:**
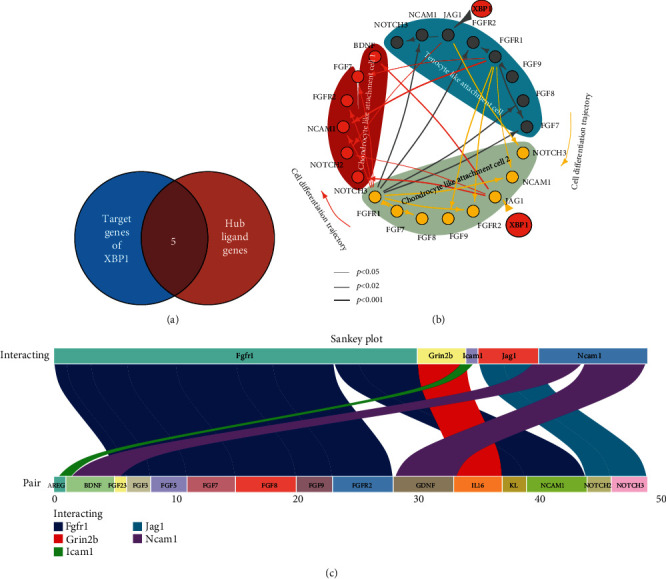
Ligand-receptor-based analysis revealed that Xbp1 promotes transcriptional regulation of this ligand gene-mediating receptor regulation of tenocyte-like attachment cell, chondrocyte-like attachment cell 2, and chondrocyte-like attachment cell 1 phenotypes. (a) Intersection analysis suggested that five ligand genes, namely, Fgfr1, Grin2b, Icam1, Jag1, and Ncam1, are potential target genes of Xbp1. (b) A Sankey plot of the potential receptor genes of these five ligand genes: Fgfr1, Grin2b, Icam1, Jag1, and Ncam1. (c) XBP1 mediates the regulation of receptor genes by promoting the transcriptional regulation of five ligand genes (namely, Fgfr1, Grin2b, Icam1, Jag1, and Ncam1) to regulate tenocyte-like attachment cell, chondrocyte-like attachment cell 2, and chondrocyte-like attachment cell 1 phenotypes.

Cellular communication is an important pathway for inducing cell differentiation. We hypothesized that XBP1 may affect intercellular communication by regulating ligand transcription. To investigate the commonalities and differences in cellular signaling exchange for each cell subtype, the CellPhoneDB was used to infer intercellular communication. Based on the results of the CellPhoneDB analysis, five ligand genes (i.e., Fgfr1, Grin2b, Icam1, Jag1, and Ncam1) were identified as potential target genes of Xbp1 (Figure 9(a)). The Sankey plot was used to demonstrate the potential receptor genes (including AREG, BDNF, FGF23, FGF3, FGF5, FGF7, FGF8, FGF9, and FGFR2) for the five ligand genes (i.e., Fgfr1, Grin2b, Icam1, Jag1, and Ncam1), GDNF, IL16, KL, NCAM1, NOTCH2, and NOTCH3. Accordingly, the XBP1 functional pattern was mapped (Figure 9(c)). This suggests that XBP1 mediates the regulation of tenocyte-like attachment cell, chondrocyte-like attachment cell 2, and chondrocyte-like attachment cell 1 phenotype by promoting the transcriptional regulation of these five ligand genes, namely, Fgfr1, Grin2b, Icam1, Jag1, and Ncam1, by receptor genes. The Pearson correlation analysis based on the GTEX database also revealed that XBP1 was significantly and positively correlated (*p* < 0.05) with the expression of JAG1 (A), NOTCH2 (B), and NOTCH3 (C) (Supplementary Figure 9A-9C). Because NOTCH2 and NOTCH3 are key effectors of the Notch signaling pathway, we proposed that Xbp1 may upregulate the Notch signaling pathway through Jag1 transcription.

**Figure 10:**
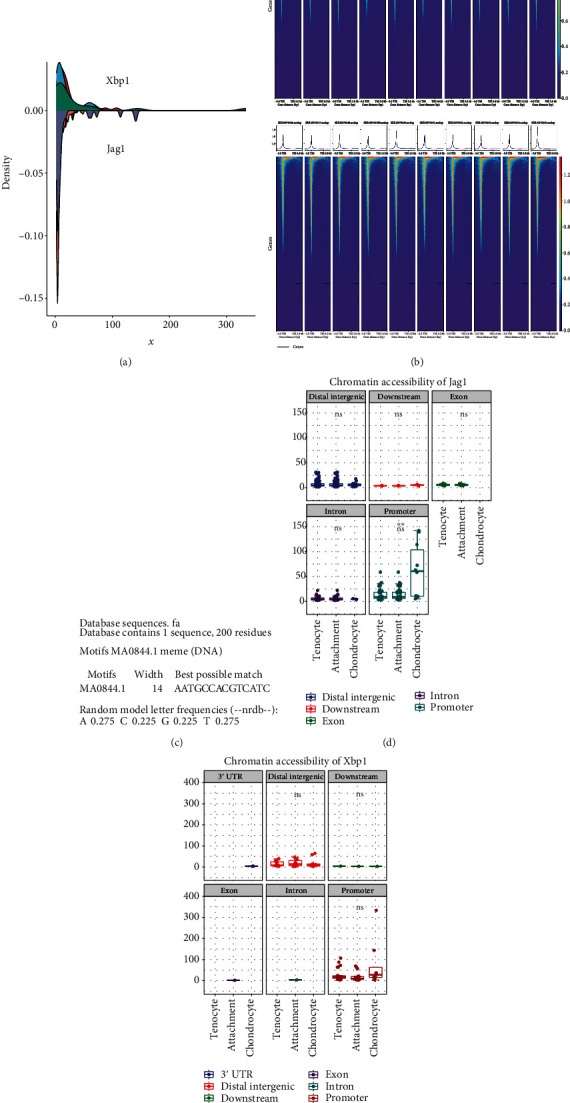
ATAC-seq-based analysis reveals that Xbp1 promotes Jag1 transcription. (a) Densitometric analysis of different transcriptional open lengths of Xbp1 and Jag1 in the tendon fibrocartilaginous region. (b) Heat map of chromatin opening analysis of different samples from the fibrocartilaginous region of the tendon. (c) Xbp1 binds to the promoter site of Jag1. (d) Chromatin accessibility of Jag1. (e) Chromatin accessibility of Xbp1.

GSE144306 and GSE168153 are datasets of the same study, and GSE144306 is an ATAC-seq dataset belonging to the tendon coupling region. The different transcriptional open lengths of Xbp1 and Jag1 in the cartilaginous region of tendon fibers are shown in Figure 10(a). This suggests that Xbp1 is common chromatin developed in different samples, while chromatin opening of Jag1 is significantly heterogeneous in GSE144306. A global analysis of GSE144306 was also performed, and a heat map was drawn for the analysis of chromatin opening in different samples of the fibrocartilaginous region of the tendons (Figure 10(b)). As a result of the ATAC-seq analysis, the MEME Suite database predicted that the promoter region of Jag1 might be regulated by Xbp1 [58, 70]. The promoter of Jag1 was significantly more open in the chondrocyte region (Figure 10(d)). By contrast, Xbp1 did not show this phenomenon (Figure 10(e)). This suggests that the transcriptional activity of Jag1 is significantly upregulated in the chondrocyte region, which may be due to the promoter activation of Jag1. Taken together, we revealed that Xbp1 might upregulate the Notch signaling pathway by promoting Jag1 transcription based on the ligand-receptor analysis and ATAC-seq analysis.

### 3.9. Crosstalk in the Notch/Hh Signaling Pathway Is Associated with Xbp1 Expression

**Figure 11:**
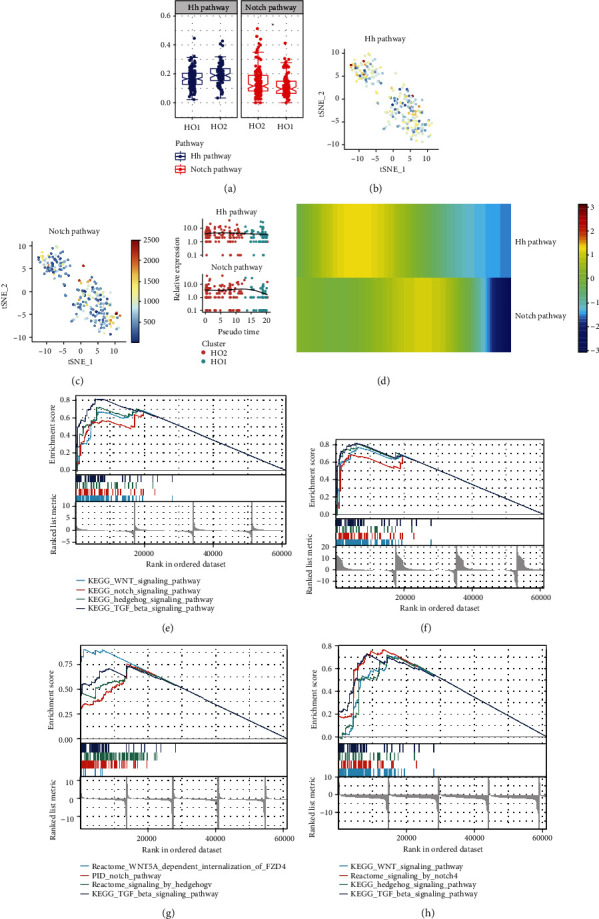
Crosstalk in the Notch/Hedgehog (Hh) signaling pathway is associated with Xbp1 expression in ectopic ossified cells. (a) Box line diagrams responding to the difference in the activities of the Hh and Notch signaling pathways in different tissues. (b) Hh signaling pathway activity in mouse tail tendon cells under simulated heterotopic ossification (HO) pathological conditions is indicated by different colors. (c) Notch signaling pathway activity in mouse tail tendon cells under simulated HO pathological conditions is indicated by different colors. (d) Proposed temporal expression characteristics of the Hh and Notch signaling pathways. (e–h) Gene Set Enrichment Analysis of XBP1 differences concerning pathway activity in different datasets: GSE94683 (e), GSE102929 (f), GSE168153 (g), and GSE138515 (h).

The GSVA based on GSE94683 revealed that upregulated activities of Hh and Notch signaling pathway activities were upregulated in HO tissues (Supplementary Figure 9D, 9E). Interestingly, the activity of the Hh signaling pathway was more expressed in HO2 than in HO1, and the activity of the Hh signaling pathway was more expressed in HO1 than in HO2 (Figures 11(a)–11(c)). By contrast, the proposed temporal expression profile of the Hh and Notch signaling pathways suggests that the Hh signaling pathway may be activated before the Notch signaling pathway. The GSEA of XBP1 expression concerning the pathway activity in different datasets was also performed (Figures 11(e)–11(h)). The upregulations of Xbp1 in GSE94683, GSE102929, GSE168153, and GSE138515 were associated with the upregulation of the WNT, Notch, Hh, and TGF beta signaling pathways. This suggests that Xbp1 upregulation may induce the occurrence of ectopic ossification by upregulating the activities of WNT, Notch, Hh, and TGF beta signaling pathways.

### 3.10. Screening for Potential MicroRNAs That Induce Upregulation of XBP1 Expression after Acute Ischemic Stroke

**Figure 12:**
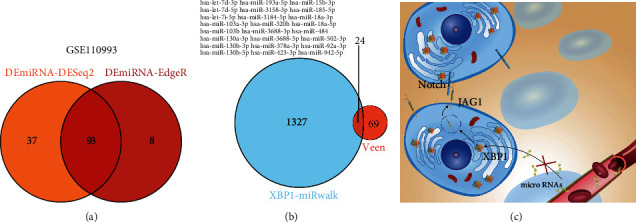
Screening for potential microRNAs causing XBP1 upregulation after acute ischemic stroke. (a) A total of 93 microRNAs were identified as differentially expressed in acute ischemic stroke by both DEseq2 and EdgeR methods that screened the GSE110993 dataset using a Venn diagram. (b) A total of 24 microRNAs that potentially target XBP1 were screened based on the miRwalk dataset. (c) Schematic depiction of this work.

Neurogenic heterotopic ossification is a common complication of acute ischemic stroke; however, its exact mechanism remains unknown. A total of 93 microRNAs have been identified as differentially expressed in acute ischemic stroke by both DEseq2 and EdgeR methods that screened the GSE110993 dataset using a Venn diagram (Figure 12(a)). In this study, the target XBP of miRNAs was predicted using the miRWalk database (http://mirwalk.umm.uni-heidelberg.de/). Based on the miRwalk dataset, we screened 24 microRNAs that could potentially target XBP1. These microRNAs included hsa-let-7d-3p, hsa-let-7d-5p, hsa-let-7i-5p, hsa-miR-103a-3p, hsa-miR-103b, hsa-miR-130a-3p, hsa-miR-130b-3p, hsa-miR-130b-5p, hsa-miR-15b-3p, hsa-miR-185-5p, hsa-miR-18a-3p, hsa-miR-18a-5p, hsa-miR-193a-5p, hsa-miR-3158-3p, hsa-miR-3184-5p, hsa-miR-320b, hsa-miR-3688-3p, hsa-miR-3688-5p, hsa-miR-378a-3p, hsa-miR-423-3p, hsa-miR-484, hsa-miR-502-3p, hsa-miR-92a-3p, and hsa-miR-942-5p (Figure 12(b)). Schematic depiction of this work was shown in Figure 12(c).

## 4. Discussion

In this study, we used a single-cell approach to integrate and analyze the developmental lineage of the fibrocartilaginous zone of tendons and the ectopic ossification cell model. Ectopic ossified cells demonstrated similar transcriptional characteristics to cells in the fibrocartilaginous region of tendons. This study also identified Xbp1 as a common key TF for ectopically ossified cells and cells in the fibrocartilaginous zone of the tendons. To the best of our knowledge, this is the first study to propose that Xbp1 may upregulate the Notch signaling pathway by transcribing Jag1. These results also suggest a potential association of endoplasmic reticulum stress with tendon bone healing and HO.

This study was performed based on the integration analyses of scRNA-seq, bulk RNA-seq, and ATAC-seq data. The CCA in the Seurat package was used for dataset integration, whereby we found that ectopically ossified cells had a similar transcriptional profile to cells in the fibrocartilaginous region of the tendons. Based on the pseudo-temporal trajectory analysis of Monocle 3, this study revealed a potential model of PC differentiation in ectopically ossified tissues. To identify specific TFs associated with ectopic ossification, we used a modified version of the SCENIC method to construct a regulatory network between HO-related targets and TFs. The results showed that Xbp1 was a common key TF for ectopically ossified cells and cells in the fibrocartilaginous region of the tendons. We then performed cellular ligand-receptor analysis using the CellPhoneDB database and software according to the instruction manual, and after further pathway screening, Xbp1 was found to possibly upregulate the Notch signaling pathway by promoting Jag1 transcription. GSE94683 (including nine samples of healthy donors and seven samples of ectopically ossified tissues from patients with HO) was subjected to differential analysis. Five TFs (i.e., GLI1, EN1, XBP1, SHOX, and TBX5) and some KEGG pathways (i.e., WNT, Notch, Hh, and TGF beta signaling pathways) were significantly highly expressed in HO tissues.

We first classified pathological cells in the ectopic ossification model into HO1 and HO2 cell subpopulations. The proposed chronological analysis suggests that HO2 is a precursor cell of HO1. Mgp, Bgn, Sox9, Col11a1, Col1a1, Col1a2, Tnmd, Col11a2, Scx, Hivep2, Bcl2, Col2a1, Mkx, Myc, and Dcn were enriched in HO2 cells. Sox9 and Scx were key TFs in the migrating connective tissue between the tendon and bone [34, 71]. Diabetes was found to contribute to the development of tendinopathy through the downregulation of Scx, Mkx, Col1a1, Col1a2, and Bgn [72], and the upregulation of Col1a1, Scx, and Dcn contributed to the control of tendinopathy [73]. Tnmd was expressed at high levels in tendon cells and ligament cells and was found to have great potential in the treatment of tendon repair, which might be related to its coordinated expression of Col2a1 synergistically promoting angiogenesis [74–76]. Mkx controlled the differentiation of tendon and ligament cells, while Bcl2 was associated with the apoptosis and regeneration of tenocytes [77, 78]. Col5a1, Foxc1, Gli3, Gli2, Pecam1, Klf2, Klf4, Zbtb48, Ptch2, Smad7, Sufu, Igfbp5, Tcf, Fbn1, and Jag1 were mainly enriched in HO1 cells. Gli2/3, Tcf, Fbn1, and Col5a1 were molecular markers in cartilage tissues [79–81]. The upregulation of Klf2 and Klf4 was found to promote cartilage stability and resist osteoarticular oxidative stress conditions [82, 83]. Foxc1, Ptch2, and Sufu were found to be essential for the activation of the Indian Hh signaling pathway [84, 85]. Smad7 and Jag1 were found to affect cartilage development through the MAPK or Notch signaling pathway [86, 87]. Igfb5 expression was found to be highly concentrated in developing limb muscle tissue and was highly correlated with osteogenesis [88]. The expression profiles of HO1 and HO2 cells were more similar to that of chondrocytes and tenocyte-like cells, respectively. Some information also implied the developmental potential of HO2 cells to become HO1 cells. For example, (1) Sox9, Col1a1, and Col2a1 have been shown to play important functions in cartilage development [89, 90]; (2) Mgp was found to be related to the onset of osteoarthritis during cartilage development [91]; (3) Bcl2 was a key protein of the PI3K-AKT signaling pathway, which was closely associated with chondrocyte proliferation [92]; and (4) COL5A1 polymorphism was associated with susceptibility to tendon disease [93]. Notably, *Gli3*, *Cli2*, *Pecam1*, and *Ptch2* were found to be the star genes of the Hh signaling pathway, and *Notch2* and *Jag1* are the star genes of the Notch signaling pathway, suggesting that the activity of the Notch and Hh signaling pathways may be upregulated in HO1 cells [94–97].

In this study, we performed an integrative analysis of single-cell transcriptome data of mouse Achilles tendon, single-cell transcriptome data of mouse tendon junction region, and single-cell transcriptome data of mouse tail tendon cells under simulated HO pathological conditions (including GSE168153, GSE138515, and GSE102929 datasets under simulated HO pathological conditions) was also performed in this study. In Figure 6(b), tenocyte-like attachment cells (Matn4 and Col2a1 are cell markers of this cell type) were found to have more HO2 cells, while more HO1 cells were found in chondrocyte-like attachment cell 1 (Anxa2 and Col14a1 are cell markers of this cell type). Matn4 and Col2a1 are cell markers of tenocyte-like attachment cells. Col2a1 is a crossover gene located between tenocyte and chondrocyte differentiation [76, 90, 98, 99]. While Matn4 expression was tightly regulated and served as a marker of cell differentiation, it played a key role in maintaining the stability of articular cartilage [100]. Anxa2 and Col14a1 are cell markers of chondrocyte-like attachment cell 1. The upregulation of Anxa2, a biomarker of cartilage development, may promote tendon healing [101, 102]. The results showed that Col14a1 was tendon-associated collagen and was associated with the development of osteoarthritis [103]. These data suggest that ectopically ossified cells have similar transcriptional and developmental characteristics to cells in the fibrocartilaginous zone.

In this study, we first identified the endoplasmic reticulum (ER) stress effector Xbp1 as a common TF for HO bulk-RNA-seq, HO scRNA-seq, and the integrated scRNA data (including GSE168153, GSE138515, and GSE102929 datasets under simulated HO pathological conditions) using a comprehensive network analysis combined with a modified SCENIC approach. XBP1 was found to be an important regulatory pathway for the unfolding protein response that occurred in response to ER stress and played an important role in both physiological and pathological conditions, and its activity had a significant effect on the progression and prognosis of numerous diseases [104]. In this study, 160 genes were identified as core downstream regulatory targets of XBP1, with significant interactions among them, and they mainly played important roles in the Golgi vesicle transport, ER protein processing, cellular communication, and translocation. Lei et al. found that mechanical stress can activate the ER stress and MAPK signaling pathways in the posterior longitudinal ligament, which played an important role in the onset of ossification of the posterior longitudinal ligament [105, 106]. The effect of ER stress on cartilage differentiation is regulated by the microenvironment [107]. Recent studies have shown that ER stress regulates the Notch and Hh signaling pathways [108, 109]. As a key effector protein of ER stress, Xbp1 is positively correlated with the activity of the Notch signaling pathway. The upregulation of Xbp1 was found to inhibit the Notch pathway effector protein pecanex, resulting in ER enlargement [110–112]. In addition, the deletion of XBP1 signaling was found to cause chondrodysplasia and delayed ossification [113]. In the present study, Xbp1, a key effector protein of ER stress, was found to be a key TF for the onset of ectopic ossification. These results also suggest a potential association between endoplasmic reticulum stress and tendon bone healing and HO.

Cellular communication is an important process for inducing cell differentiation. We hypothesized that XBP1 may affect intercellular communication by regulating the transcription of ligands. To investigate the similarities and differences in intercellular information exchange for each cell subtype, we used the CellPhoneDB to infer intercellular communication and constructed a functional model of XBP1 regulation accordingly. We propose that Xbp1 may upregulate the Notch signaling pathway through Jag1 transcription. Based on the results of the chromatin opening analysis, the MEME Suite database predicted that the promoter region of Jag1 might be regulated by Xbp1. ER stress was found to upregulate Jag1 expression, while the relationship between Xbp1 and Jag1 has not been elucidated. To our knowledge, this study is the first to propose that Xbp1 may upregulate the Notch signaling pathway by promoting Jag1 transcription [114]. In GSE94683, GSE102929, GSE168153, and GSE138515, the upregulation of Xbp1 was found to be associated with the upregulation of WNT, Notch, Hh, and TGF beta signaling pathways, which may be due to the crosstalk of these pathways in ectopic ossified tissues. However, the specific regulatory mechanisms remain to be further investigated [7, 115, 116].

The prevalence of HO in stroke is 0.5%-1.2%, which has led to a lack of understanding of the epidemiology and pathogenesis of HO after ischemic stroke [117–119]. Furthermore, the occurrence of HO is dependent on the upregulation of endoplasmic reticulum stress pathways [105, 106, 120–122]. Meanwhile, the activation of endoplasmic reticulum stress-related pathways and the upregulation of XBP1 are key features of the widespread presence of various brain-related diseases, including stroke [123]. Therefore, the coupregulation of endoplasmic reticulum stress-related pathways in HO and ischemic stroke has attracted our attention. The current study found that the expression of microRNAs carried by exosomes in the blood is altered after ischemic stroke, which may affect the function of organs and tissues in the distant compartment [124–126]. We propose the hypothesis that changes in microRNA levels in the peripheral blood exosomes following ischemic stroke may lead to upregulation of stem cell XBP1 expression in muscle tissue and may thus induce neurogenic ectopic ossification. In this study, 24 microRNAs were screened and were found to be potentially associated with the upregulation of XBP1 expression after acute ischemic stroke. However, the effect of circulating blood exosomes on the occurrence of HO remains to be further investigated.

In this study, cell clusters of ectopic ossification phenotypes were isolated in a single-cell dataset of ectopic ossification. Three single-cell transcriptomes (namely, fibrocartilaginous zone single cell, Achilles tendon single-cell transcriptome, and ectopic ossification model transcriptome) were integrated into one dataset using the CCA method. Ectopically ossified cells were found to colocalize with the fibrocartilaginous zone cells, suggesting that ectopically ossified cells share similar transcriptome characteristics to fibrocartilaginous zone cells. The pseudo-temporal trajectory analysis further supported the above findings. Further TF coexpression network analysis revealed that Xbp1 is a hub TF for HO formation. The ligand-receptor analysis reveals potential pathways through which Xbp1 exerts its transcriptional regulatory role. The single-cell analysis revealed that the Xbp1 and Notch pathway expressions were upregulated in ectopic ossified tissues, and they were positively correlated with each other. Ultimately, we proposed a potential mechanism by which Xbp1 may upregulate the Notch signaling pathway by promoting Jag1 transcription. In summary, this study integrated six datasets, namely, GSE94683, GSE144306, GSE168153, GSE138515, GSE102929, and GSE110993. The differentiation trajectory and key TFs of ectopic ossification occurrence were systematically analyzed by integrating scRNA-seq, bulk RNA-seq, and ATAC-seq data of the fibrocartilaginous region and HO.

This study revealed similarities between ectopically ossified cells and cells of the fibrocartilaginous zone of tendons and suggested a potential Xbp1/Jag1/Notch signaling pathway. However, a theoretical work with data from databases with no experimental verification may limit the application value of this research. Further functional experiments are still needed to be performed to explore the specific functional mechanisms of Xbp1. In this study, we obtained data from an integrated analysis of multiple databases and planned to conduct further analysis using ectopic ossified tissues and tendon fibrocartilaginous zone cells from the same model. In addition, the regulatory relationships between the Hh and Notch signaling pathways and other key pathways need to be further validated to confirm the activation patterns of the pathways in the single-cell transcriptome. We believe that further collection of clinical samples is also necessary, which will help in clinical diagnosis and translation.

## 5. Conclusion

A systematic analysis of the differentiation landscape and cellular homogeneity facilitates a molecular understanding of the potential homology between tendon fibrocartilage and ectopic ossified tissue. Furthermore, by identifying Xbp1 as a hub regulator and through ligand-receptor analysis, we propose a potential Xbp1/Jag1/Notch signaling pathway. The findings provide a novel concept for the formation of ectopically ossified tissues, i.e., HO cells may be differentiated from misplaced fibrocartilaginous zone stem cells.

## Figures and Tables

**Table 1:** Raw data working sheet.

Mesenchymal stromal cells in ectopic ossified tissues	GSE94683 from GEO database
A single-cell sequencing dataset available from the fibrocartilaginous region of mouse tendons	GSE144306 from GEO database
A ATAC-seq dataset available from the fibrocartilaginous region of mouse tendons	GSE168153 from GEO database
A scRNA-seq dataset from the Achilles tendon of 6-week-old male C57Bl/6 mice	GSE138515 from GEO database
A scRNA-seq dataset from 4-week-old mouse tail tendon cells	GSE102929 from GEO database
A dataset reflecting microRNA changes after acute ischemic stroke	GSE110993 from GEO database
Datasets used to screen for differentially expressed TF in HO tissue	The TTRUST dataset

## Data Availability

The corresponding author agreed to provide the reader with the original data for a suitable reason.

## Supplementary Materials

Supplementary Materials All supplementary figures were included in the “Supplementary figures” file.
