# Supplementary material for: Single-Cell Integration Analysis of Heterotopic Ossification and Fibrocartilage Developmental Lineage: Endoplasmic Reticulum Stress Effector Xbp1 Transcriptionally Regulates the Notch Signaling Pathway to Mediate Fibrocartilage Differentiation
Source: Oxid Med Cell Longev. 2021 Oct 26;2021:7663366. doi: 10.1155/2021/7663366 (PMC8563124; doi:10.1155/2021/7663366)
Supplement: Supplementary Materials — All supplementary figures were included in the “Supplementary figures” file. [file 7663366.f1.doc]

**Supplementary Figures**

**
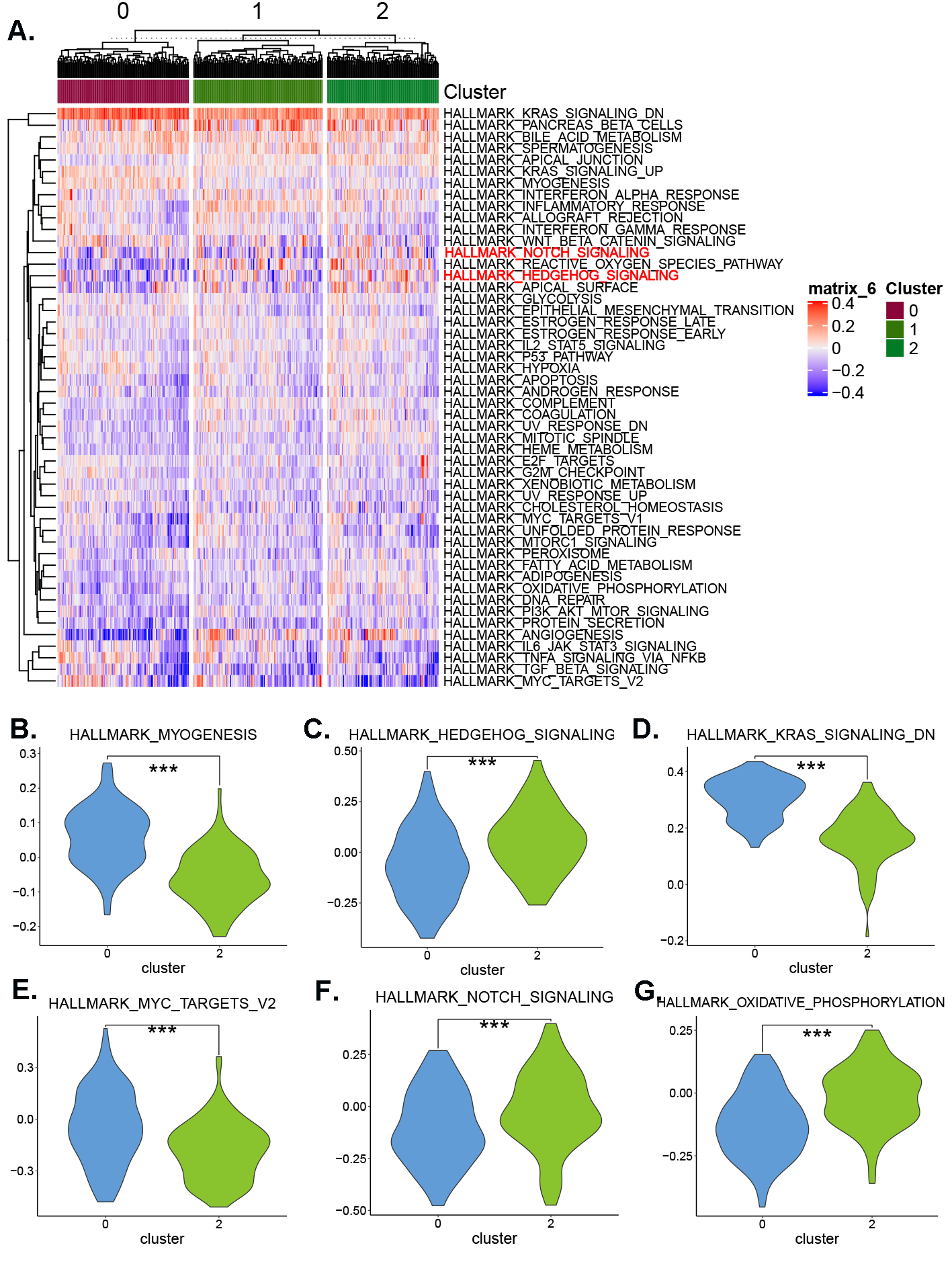
**

Supplementary Figure 1. Heterogeneity of signaling pathways in different cell subpopulations in mouse tail tendons under physiological and simulated heterotopic ossification (HO) conditions.

A.Differential expression of signaling pathways in different cell subpopulations in mouse tail tendons under physiological and simulated HO pathological conditions.

B-G. Differential activation of pathways between cell subpopulation clusters 0 and 2 in mouse tail tendons under physiological and simulated HO pathological conditions.


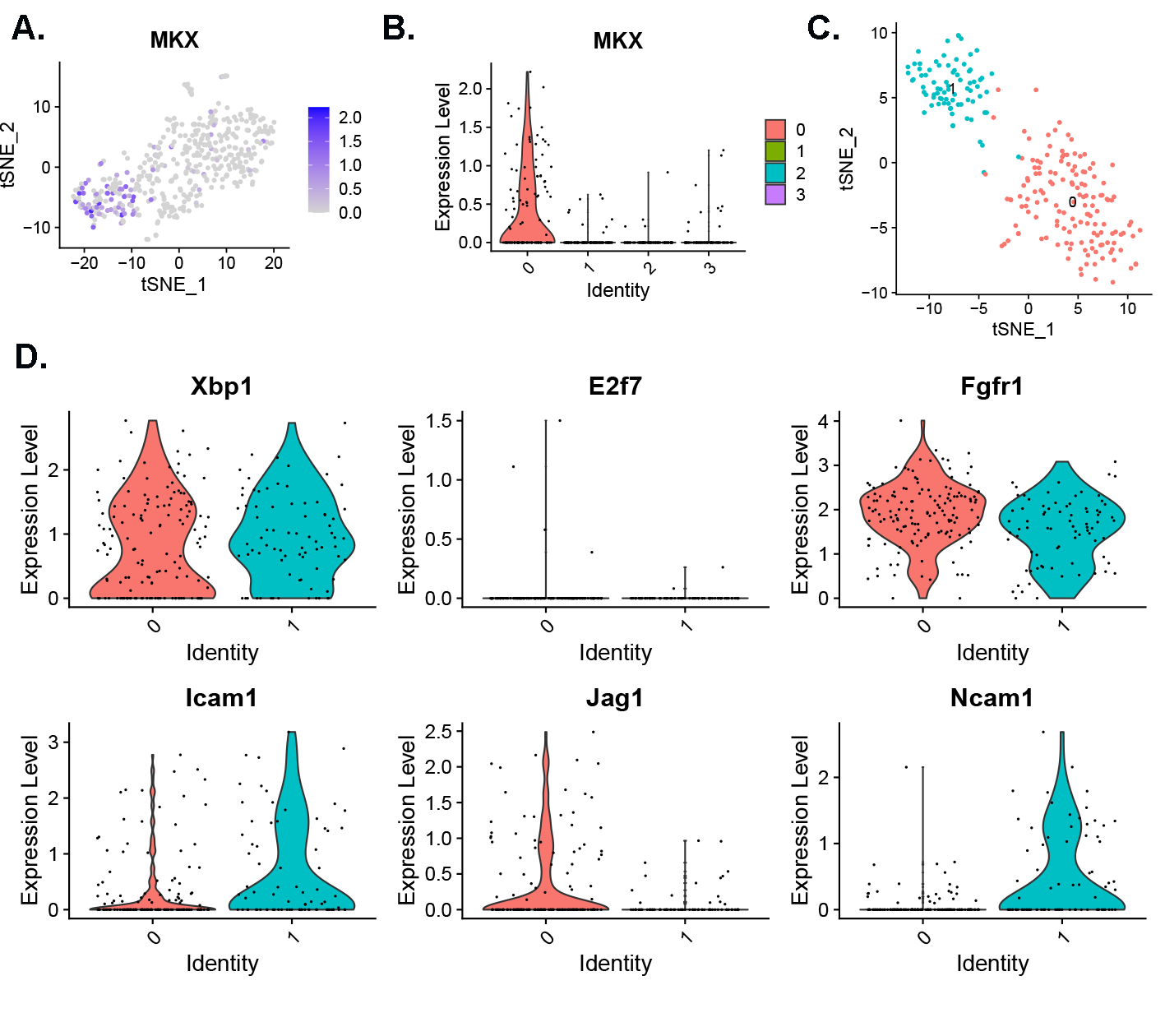


Supplementary Figure 2. Characterization of the expression of key genes in mouse tail tendons under simulated heterotopic ossification (HO) pathological conditions.

A-B. The t-distributed stochastic neighbor embedding (tSNE) subgroup clustering maps of mouse tail tendons under physiological and simulated HO pathological conditions were colored, and bar graphs were drawn based on the relative expression intensity of Mkx.

C. Clustering of tSNE subgroups in mouse tail tendons under simulated HO pathological conditions, with different clusters indicated by different colors.

D. Differential expression of six genes (Xbp1, E2f7, Fgfr1, Icam1, Jag1, and Ncam1) in mouse tail tendons under simulated HO pathological conditions in different cell subpopulations.


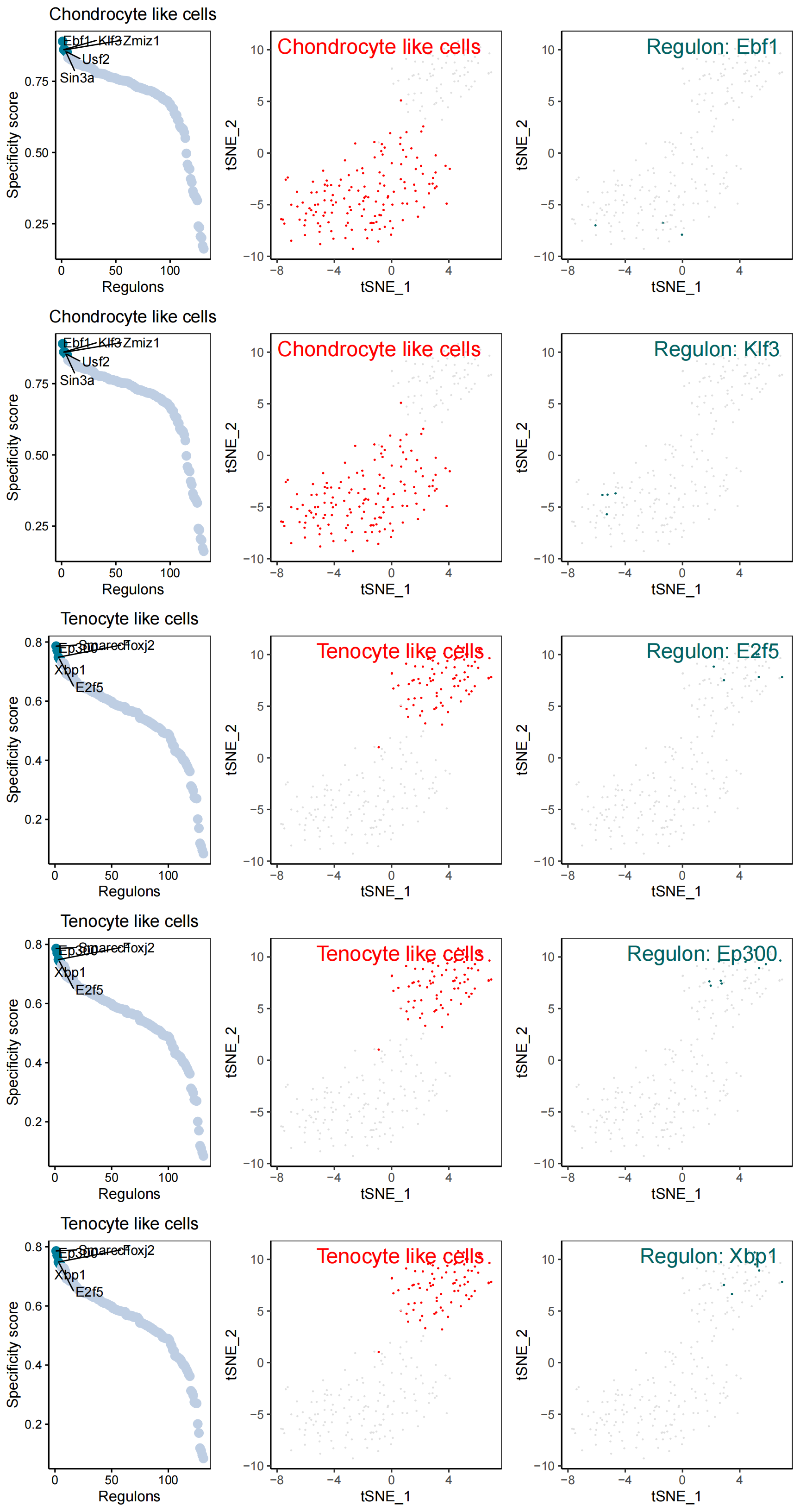


Supplementary Figure 3. Analysis of cell type-specific transcription factor (TF) activity. The key TFs in different cell subpopulations in mouse tail tendons under simulated heterotopic ossification (HO) pathological conditions were sorted, and the degree of expression of the transcription factors was marked with t-distributed stochastic neighbor embedding (tSNE) plots.


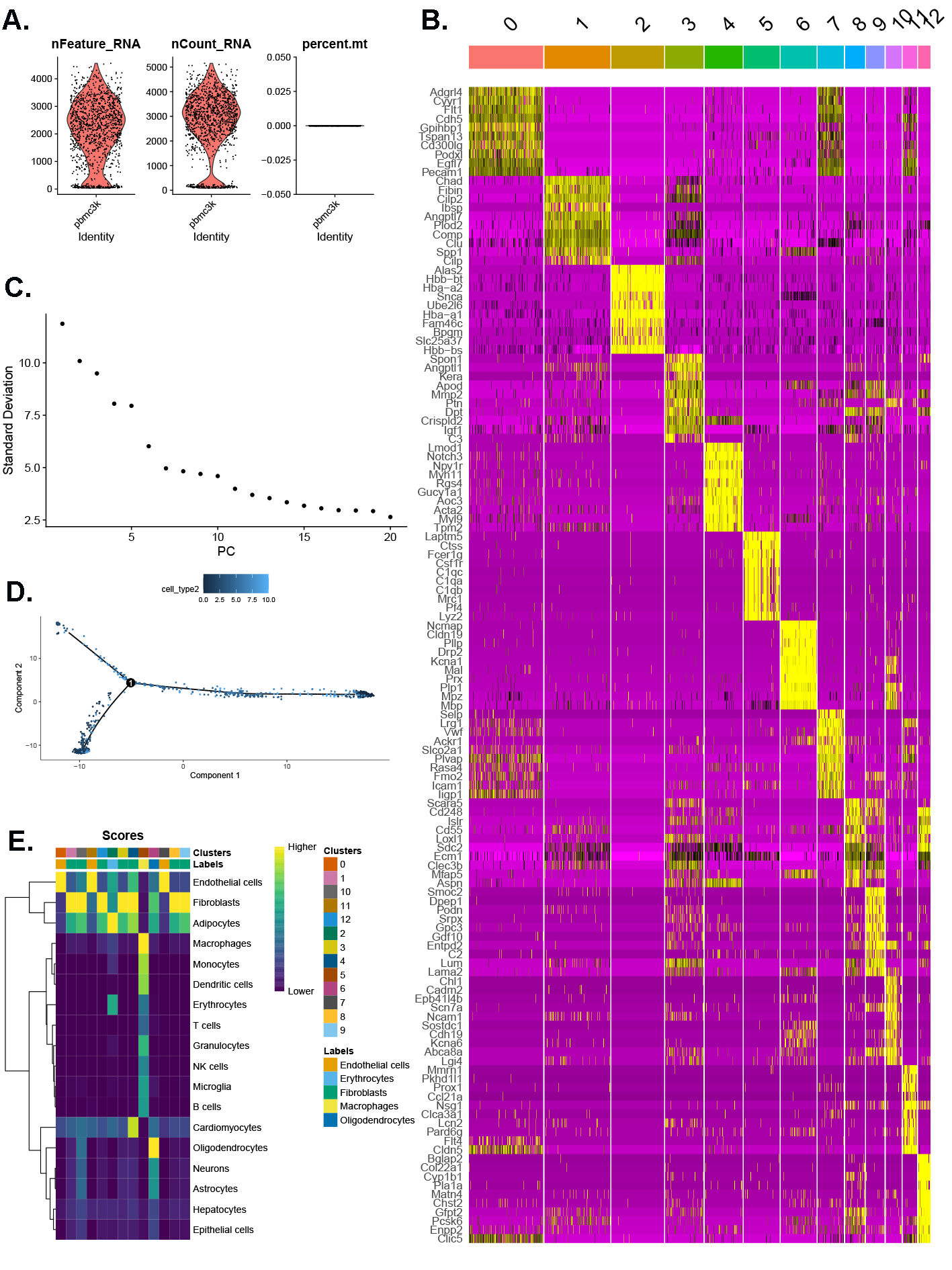


Supplementary Figure 4. Cellular annotation and heterogeneity analysis of the GSE138515 dataset.

A. Quality control process for the GSE138515 dataset with the removal of mitochondrial mRNA.

B. Heat map showing the top 10 marker genes for each cell cluster.

C. Identification of 12 principal components (PCs) by principal component analysis (PCA).

D. Pseudo-temporal trajectory analysis shows a clear pattern of cell differentiation in tendons.

E. Heat map of cell annotation for different clusters based on the "singleR" package.


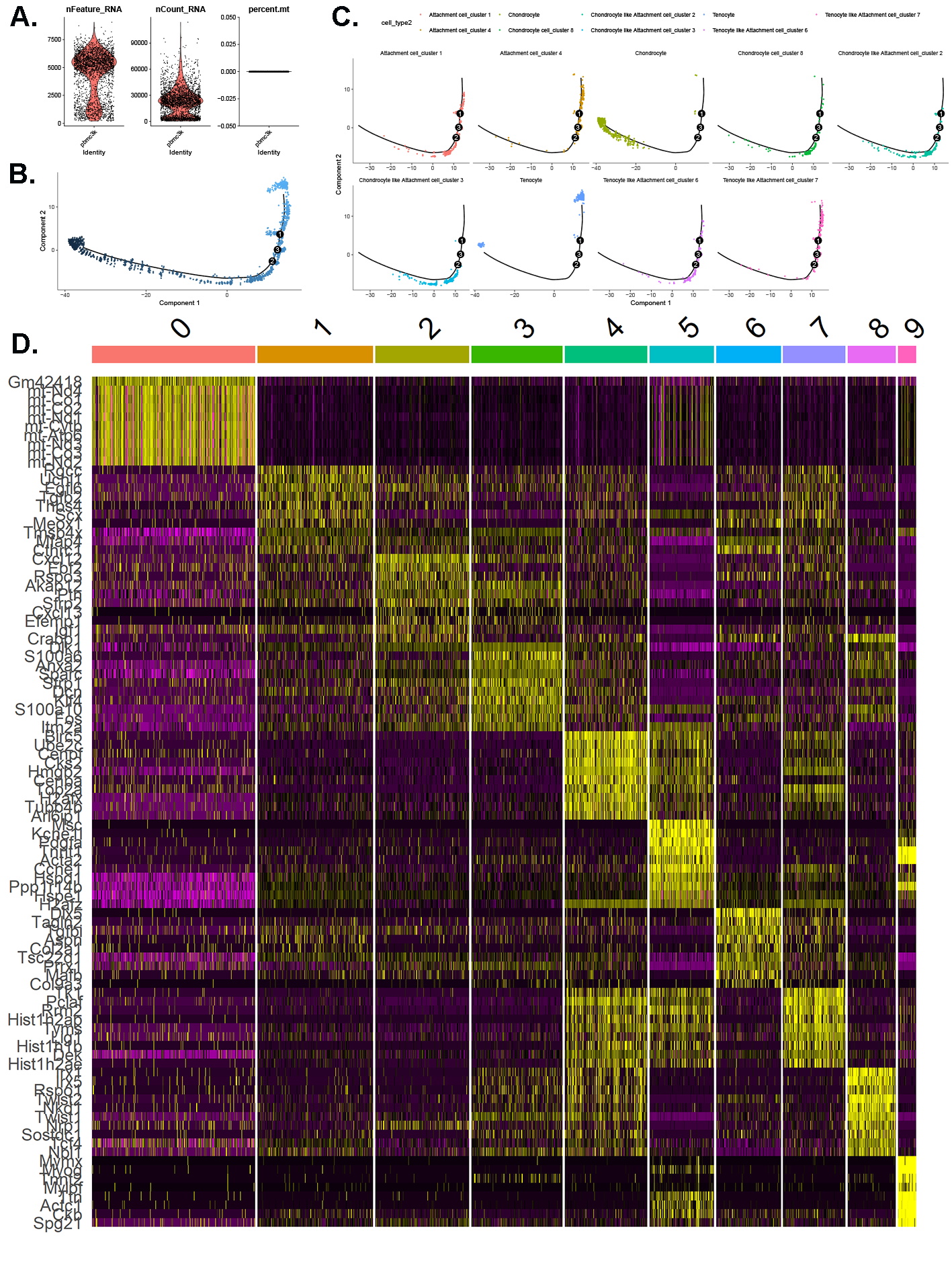


Supplementary Figure 5. Cellular annotation and heterogeneity analysis of the GSE168153 dataset.

A. Quality control process for the GSE168153 dataset with the removal of mitochondrial mRNA.

B. Pseudo-temporal trajectory analysis showing cells in tendons with distinct differentiation patterns.

C. A faceted plot of the distribution of temporal trajectories for different cell types in the GSE168153 dataset.

D. Heat map showing the top 10 marker genes for each cell cluster.


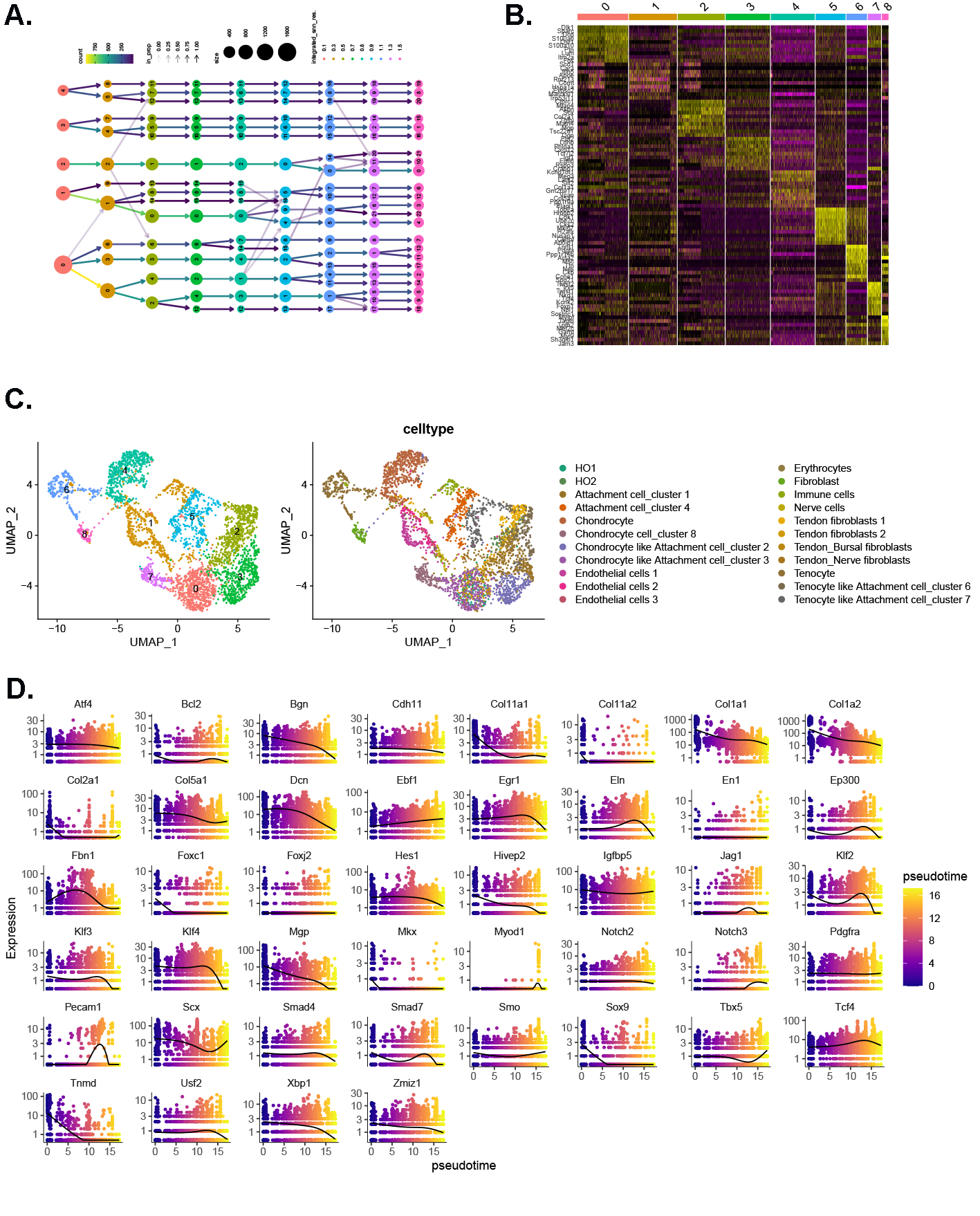


Supplementary Figure 6. Integration analysis of GSE168153, GSE138515, and GSE102929 datasets under simulated heterotopic ossification (HO) pathological conditions.


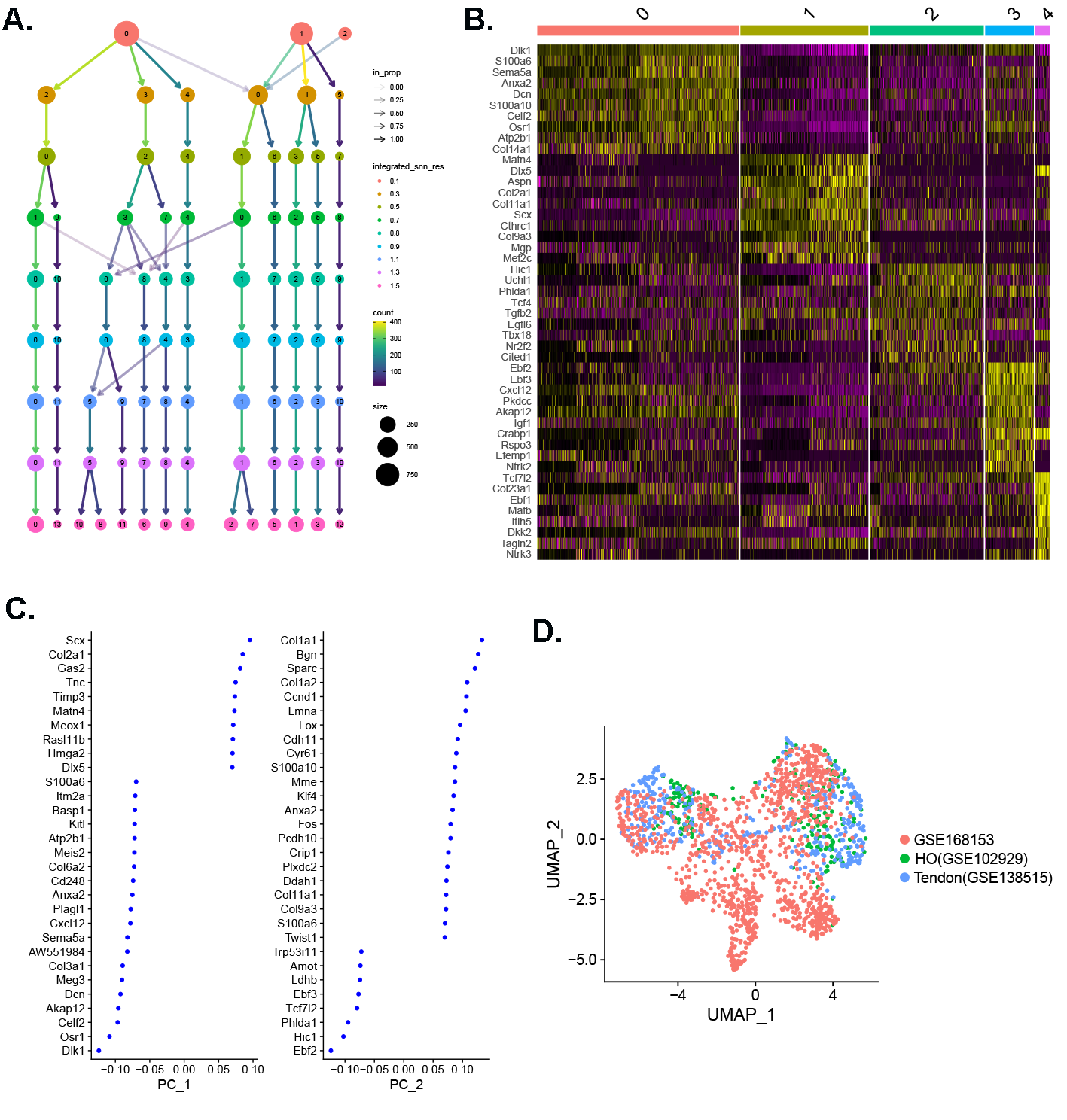


Supplementary Figure 7. Heterogeneity analysis of tenocyte-like attachment cell 1 and chondrocyte-like attachment cell 1.

A. Five principal components (PCs) were identified based on the principal component analysis (PCA) clustering dendrogram.

B. Heat map showing the top 10 marker genes for each cell cluster.

C. Marker genes of PC1 and PC2 in the integrated dataset of “tenocyte-like attachment cell 1 and chondrocyte-like attachment cell 1”.

D. Uniform manifold approximation and projection (UMAP) plot showing the distribution of tenocyte-like attachment cell 1 and chondrocyte-like attachment cell 1.


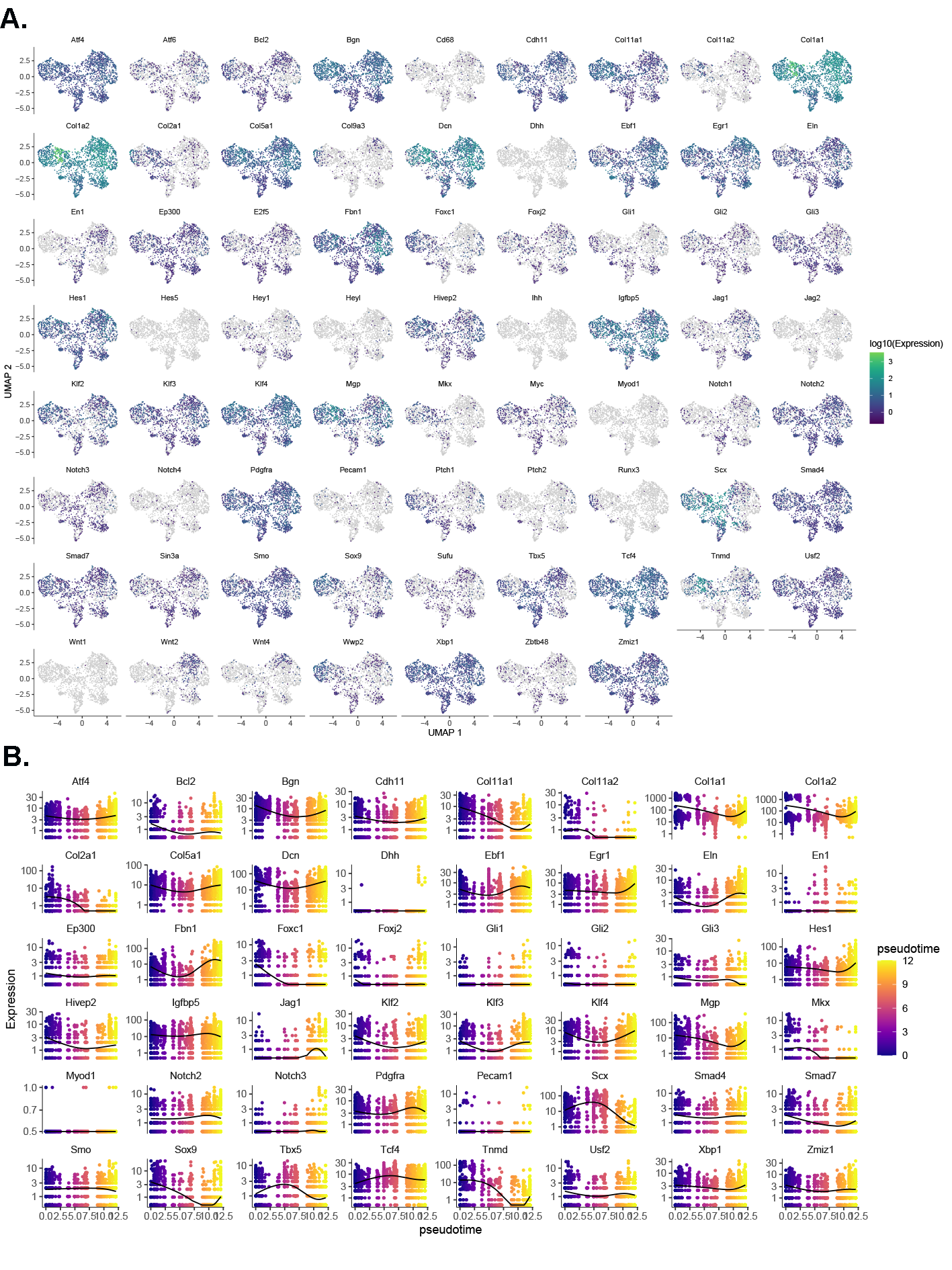


Supplementary Figure 8. Gene expression characteristics and pseudo-temporal differentiation trajectories of tenocyte-like attachment cell 1 and chondrocyte-like attachment cell 1.

A. Single-cell gene expression profiles based on “tenocyte-like attachment cell 1 and chondrocyte-like attachment cell 1”, showing the expression characteristics of genes related to ectopic ossification and the tendon fibrochondral region.

B. Expression of heterotopic ossification (HO)-related genes in “tenocyte-like attachment cell 1 and chondrocyte-like attachment cell 1” varies with differentiation chronology.


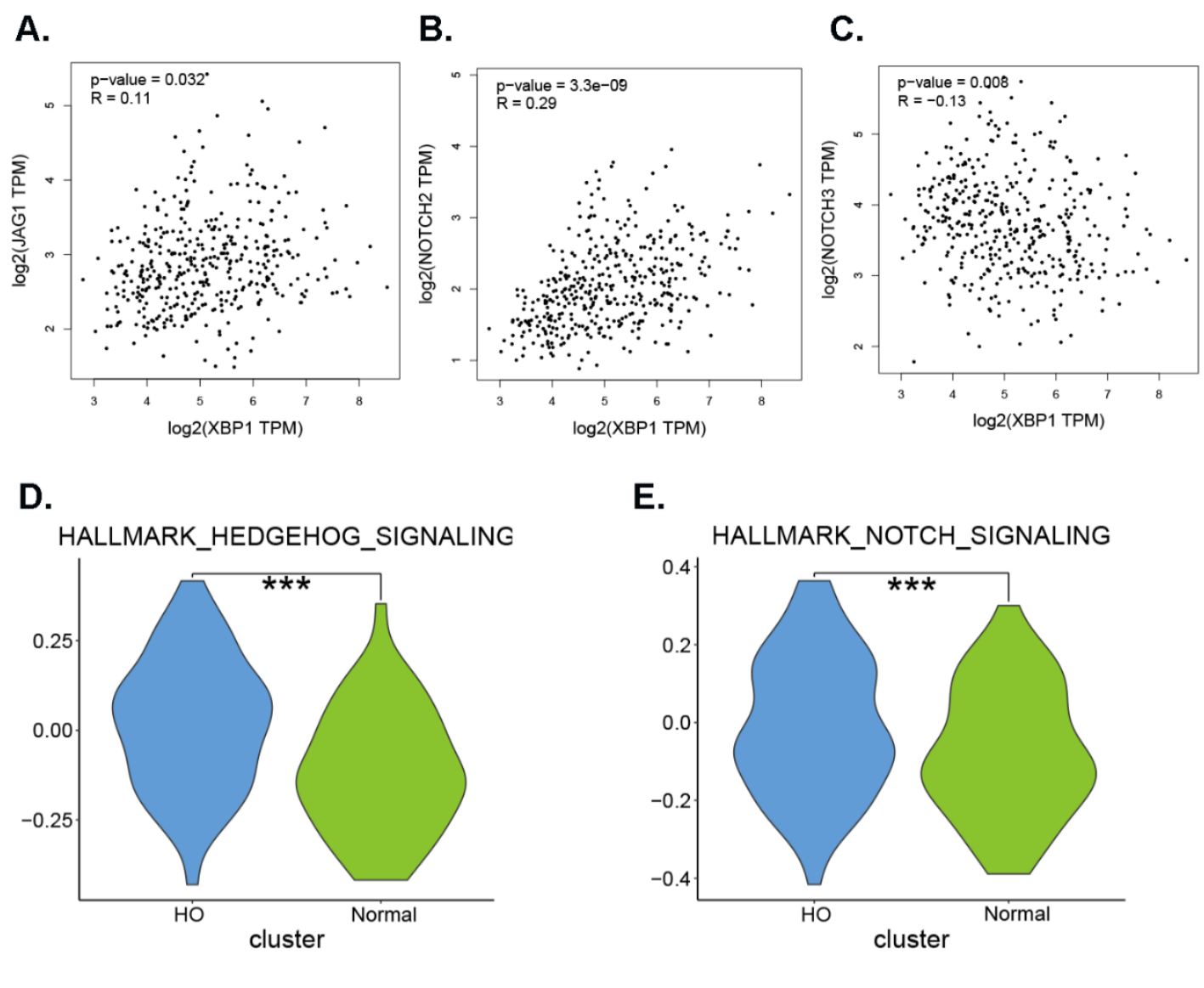


Supplementary Figure 9. The occurrence of ectopic ossification and the expression of XBP1 were associated with the activation of the Notch and Hedgehog (Hh) signaling pathways.

A-C. Person correlation analysis based on the Genotype-Tissue Expression (GTEx) database revealed that XBP1 was positively correlated with the expression of JAG1 (A), NOTCH2 (B), and NOTCH3 (C).

D-E. Gene set variation analysis for microarray and RNA-Seq data (GSVA) based on the GSE94683 dataset revealed the upregulation of the Hh and Notch signaling pathways in heterotopic ossification (HO) tissues.
